# Diet-Induced Hyperhomocysteinemia in C57BL/6 Mice: Simultaneous Evaluation of Plasma Homocysteine, Methionine, and Cysteine by LC-MS/MS and Assessment of Associated Oxidative Stress in Brain Homogenates

**DOI:** 10.3390/antiox15091162

**Published:** 2026-09-11

**Authors:** Maria Luisa Valle, Christopher Lawrence De Jesus, Bitseat Getaneh, Hediye Erdjument-Bromage, James Loveland, Christopher William, Donatello Arienzo, Thomas A. Neubert, Agueda Rostagno, Jorge Ghiso

**Affiliations:** 1 Department of Pathology, NYU Grossman School of Medicine, New York, NY 10016, USA; maria.valle@nyulangone.org (M.L.V.); bkg18@georgetown.edu (B.G.); jamespl@umich.edu (J.L.); christopher.william@nyulangone.org (C.W.); 2 Institute of Translational Neuroscience, NYU Grossman School of Medicine, New York, NY 10016, USA; clcdejesus@icloud.com (C.L.D.J.); hediye.bromage@nyulangone.org (H.E.-B.); thomas.neubert@nyulangone.org (T.A.N.); 3 Department of Psychology, San Diego State University, San Diego, CA 92182, USA; darienzo@sdsu.edu

**Keywords:** homocysteine, methionine, cysteine, biomarker quantification, liquid chromatography–mass spectrometry, oxidative stress

## Abstract

Hyperhomocysteinemia (HHcy), a risk factor for cardiovascular, neurodegenerative, and metabolic dysfunctions in humans, has been recapitulated in murine models by genetic and dietary modifications. The relevance of HHcy in clinical and experimental settings has led to the development of various analytical techniques with varying sensitivity and sample volume requirements. This study uses a fit-for-purpose, small-volume LC-MS/MS workflow to quantify plasma homocysteine (Hcy), methionine (Met), and cysteine (Cys) in a diet-induced HHcy mouse model. Using these assays that overcome limitations imposed by sample availability in small animals, our study evaluated circulating levels of these amino acids in C57BL/6 mice fed a HHcy-inducing diet rich in Met and lacking vitamins B6, B9, and B12. The increased Hcy and decreased Cys plasma concentrations observed in these mice correlated with decreased glutathione concentrations in brain homogenates and concomitant increase in the activity of the antioxidant enzyme Glutathione-S-Transferase and in the levels of the lipid peroxidation marker malondialdehyde. Overall, our work links plasma HHcy to the induction of brain oxidative stress, supporting the role of dietary modifications as complementary therapeutic strategies for neurodegenerative conditions.

## 1. Introduction

**Figure 1:**
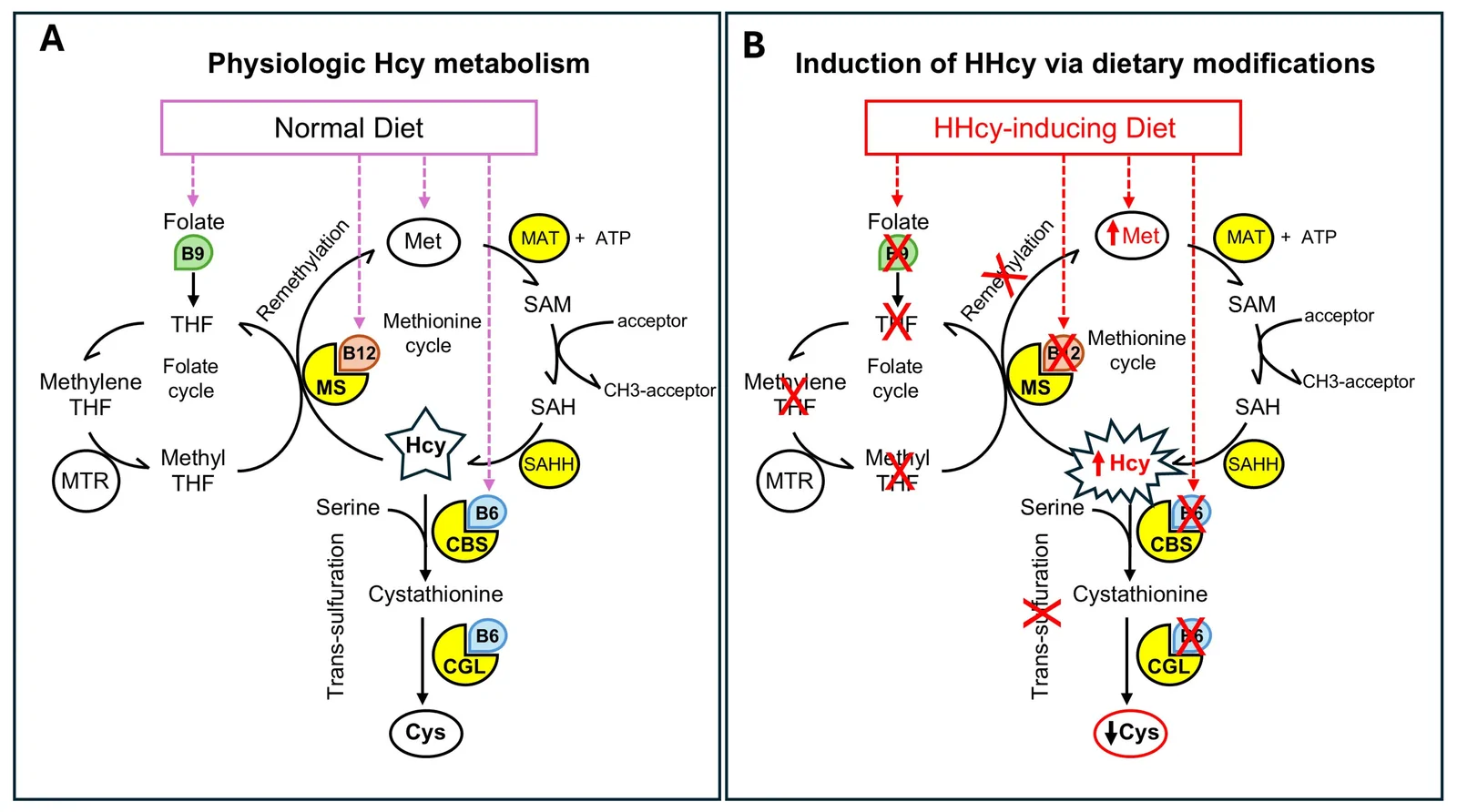
Homocysteine physiological metabolic pathways and changes induced by dietary modifications. (**A**) Under normal physiological conditions, Met is converted to SAM in the presence of MAT and ATP. In turn, SAM donates the methyl group to DNA, protein, and lipid substrates, generating SAH which is hydrolyzed by the action of SAHH and converted in Hcy. The Hcy molecule can be remethylated and converted back into Met through the enzymatic activity of MS using vitamin B12 as a cofactor. For the remethylation to occur, folic acid (vitamin B9) is needed for the synthesis of 5-methyl THF, a methyl donor for the conversion of Hcy into Met via MS. Alternatively, Hcy may be further converted into Cys through the sequential activity of CBS and CGL in the presence of vitamin B6 as a cofactor in the so-called trans-sulfuration pathway. (**B**) A diet rich in methionine and lacking B6, B9, and B12 increases the generation of Hcy and inhibits the participation of the amino acid in the remethylation and trans-sulfuration pathways, resulting in Hcy accumulation. Hcy indicates homocysteine; HHcy, hyperhomocysteine; Met, methionine; Cys, cysteine; THF, tetrahydrofolate; MTR, methylene THF reductase; MS, methionine synthase; SAM, S-adenosylmethionine; MAT, methionine adenosyl transferase; SAH, S-adenosyl homocysteine; SAHH, S-adenosylhomocysteine hydrolase; CBS, cystathionine β-synthase; CGL, cystathionine γ-lyase.

Homocysteine (Hcy) is a non-essential and non-protein-forming sulfur amino acid involved in one-carbon metabolism and in the biosynthesis of nucleotides and amino acids. Hcy originates as an intermediate in the metabolism of dietary methionine (Met) and it is situated at a branch point of a metabolic pathway in which it can be either enzymatically remethylated to generate Met, or irreversibly catabolized into cysteine (Cys) through a trans-sulfuration process [1,2]. The Met cycle is initiated by the action of methionine adenosyl transferase (MAT) on the dietary Met which is converted into S-adenosylmethionine (SAM)—the ultimate methyl donor for all methylation processes within the cell—providing the one-carbon units to biochemical reactions involving DNA, RNA, histones, and proteins, among others. During this process, SAM generates S-adenosylhomocysteine (SAH), which is further hydrolyzed to adenosine and Hcy in the presence of SAH hydrolase (SAHH). Once formed, Hcy can be recycled back to Met—the final step of the methionine cycle—by the vitamin B12-dependent enzyme Met synthetase (MS) and 5-methyl tetrahydrofolate (THF) as a methyl donor. Alternatively, as indicated above, Hcy can be catabolized via the trans-sulfuration pathway leading to the production of Cys. The first step in this pathway involves cystathionine β-synthase (CBS), an enzyme that catalyzes the condensation of Hcy and the amino acid serine to generate cystathionine, which is further hydrolyzed by cystathionine-γ-lyase (CGL) to produce Cys, with both steps dependent on vitamin B6 as cofactor (Figure 1A). The trans-sulfuration pathway links one-carbon metabolism to redox homeostasis as Cys plays a key role in the antioxidant defense, both through its free radical scavenger activity and as a precursor of glutathione, one of the most important cellular antioxidant molecules in the protection from oxidative stress-induced cellular damage [3,4,5].

Increased Hcy concentrations have been recognized as a risk factor for numerous pathological conditions including diabetes, cardiovascular diseases, and neurodegenerative disorders [1,6,7,8,9]. In humans, physiological plasma levels of the amino acid range between 6 and 10 µmol/L in men and 8 and 12 µmol/L in women [10]. Blood concentrations higher than 12 µmol/L are defined as hyperhomocysteinemia (HHcy) which, in turn, can be classified into mild (12–30 µmol/L), moderate (30–100 µmol/L), and severe (above 100 µmol/L) [1,7]. Based on their ample use and relevance in research settings, Hcy levels have also been studied in mice. Comparable to values found in humans, normal plasma levels in these laboratory animals are typically in the range of 3–5 μmol/L, although some variations have been reported in association with sex, age, and strain [11,12,13].

Elevated Hcy levels have been experimentally reproduced in murine models both by genetic manipulations of the enzymes involved in the methionine remethylation and trans-sulfuration pathways and by dietary modifications [14]. One of the most common dietary approaches to inducing Hcy accumulation relies on the administration of diets containing high concentrations of Met and lacking vitamins B6, B9, and B12 [13,14,15,16]. This diet composition, as a result of the absence of the enzymatic cofactors B6 and B12, hijacks Hcy metabolism, preventing its conversion into Met by MS or into cystathionine via CBS. The additional folic acid (vitamin B9) insufficiency in the diets also blocks the production of 5-methyl tetrahydrofolate (THF), the methyl donor in the conversion of Hcy into Met through the action of MS (Figure 1B). It has been shown that rodents administered HHcy-inducing diets recapitulate many of the structural and functional changes in the vasculature, cerebral microhemorrhagic and atherosclerotic alterations, proinflammatory vascular phenotypes, and endothelial dysfunction associated with elevated levels of the amino acid in humans [14,15,17,18,19,20,21,22]. Many of the endothelial alterations induced by HHcy have been linked to the role of the amino acid in the generation of oxidative stress conditions, which lead to the oxidation of sulfhydryl groups in endothelial nitric oxide synthase (eNOS) with concomitant reduction in nitric oxide (NO) bioavailability and subsequent vasomotor dysfunction [23,24]. The molecular mechanism associated with this process primarily involves the auto-oxidation of Hcy in the presence of transition metals—a mechanism that acquires relevance in cases of hyperhomocystinemia—leading to the generation of reactive oxygen species like ion superoxide and hydrogen peroxide [25,26]. Hcy is also linked to redox homeostasis through the transulfuration pathway. Defects in this path—induced genetically or by dietary modifications as in the current work—lead to reduction or even depletion of Cys, not only an antioxidant by itself, but also a key substrate for the synthesis of glutathione, a major intracellular antioxidant [3,4,14,27].

The relevance of elevated levels of Hcy for different pathological conditions, and the consequent need to quantitate this amino acid in clinical and experimental settings led to the development of various analytical techniques. Among them, immunoassays with colorimetric, fluorescence, and chemiluminescence read-outs have been widely used in clinical settings as a result of their automation capabilities and high throughput combined with a lack of requirement for sophisticated equipment [28,29]. Despite these advantages, immunoassays often suffer from low sensitivity, limited specificity resulting from cross-reactivity with similar compounds present in the biospecimen, particularly Cys, as well as non-specific binding and/or other sample interferences [30]. More recently, methods involving liquid chromatographic separation followed by fluorescence, ultraviolet, or mass spectrometric detection have been developed [29,31]. These methodologies, in particular high-performance liquid chromatography (HPLC) coupled with tandem mass spectrometric detection (LC–MS/MS), not only provide higher sensitivity and specificity, but they also allow the simultaneous detection of different metabolites present in the complex biological samples in wide concentration ranges [31,32]. In this sense, changes in Met and Cys levels, alone or in combination, have been studied in patients with HHcy via LC–MS/MS mainly using dried blood samples spotted onto filter paper [33,34,35]. In the limited reports that used plasma specimens, at least 50 µL samples were required for the LC-MS/MS analysis, representing a significant drawback for biological samples of limited availability or when studying blood specimens from animal models, particularly mice, in which sample acquisition may be difficult [32,36,37].

Utilizing plasma samples obtained from mice subjected to the HHcy-inducing diet described above, the present work describes an LC-MS/MS methodology that allows the simultaneous quantitative assessment of the metabolically interrelated molecules Hcy, Met, and Cys using 5-fold-lower plasma volumes than in previously described alternative techniques. Using these assays that overcome limitations imposed by sample availability in small animals, our study links the dietary-induced elevated levels of Hcy in C57BL/6 mice to decreased Cys plasma concentrations, associating these metabolic changes with reduced levels of glutathione and concomitant induction of oxidative stress conditions in brain homogenates. Overall, the studies highlight the important role of homocysteine metabolism for the maintenance of normal physiological conditions and provide insight into the relevance of considering dietary modifications and/or nutrient supplementation as essential contributors in therapeutic strategies targeting different pathological disorders.

## 2. Materials and Methods

### 2.1. Materials

L-homocysteine, L-methionine, and L-cysteine—along with the corresponding deuterated analogs L-homocysteine-d_4_, L-methionine-d_3_, and L-cysteine-d_3_—were purchased from MedChemExpress (Monmouth Junction, NJ, USA). Dithiothreitol (DTT), formic acid (FA) and trifluoroacetic acid (TFA), as well as LC-MS-grade acetonitrile and water, were obtained from Thermo Fisher Scientific (Waltham, MA, USA).

### 2.2. Animal Studies

C57BL/6 mice were purchased from Jackson Laboratories and housed at the NYU Langone School of Medicine Animal Facilities in accordance with the National Institute of Health guidelines. Mice of both genders were aged until 6 months old before being randomly assigned to either the control (CTL) (*n* = 19; 9 females and 10 males) or hyperhomocysteine (HHcy)-inducing diets (*n* = 20; 10 females and 10 males) for 16 weeks in accordance with animal protocols approved by the NYU Institutional Animal Care and Use Committee (IACUC). Both diets, custom-made from Envigo, were matched in calories and irradiated to ensure prolonged shelf-live and absence of microorganisms. The CTL food consisted of a standard rodent chow formulation with regular methionine (3.0 g/kg) and vitamins contents (TD.220641; Supplementary Table S1). The HHcy-inducing diet was rich in methionine (7.7 g/kg) and deficient in folate, vitamin B6, and B12 (TD.220642; Supplementary Table S2). Pink or yellow food coloring was added to the CTL and HHcy-inducing diet, respectively, for rapid visual distinction. Mice were housed in a temperature-controlled room with a 12/12 h light/dark cycle and ad libitum access to food and water. Animal body weight and food consumption—estimated as an average per mouse within each cage—were monitored weekly. Mice exhibiting signs of poor health conditions during the study were excluded from the analysis and euthanized. At the conclusion of the feeding experiment the remaining mice cohorts consisted of 14 controls (5 females and 9 males) and 16 animals in the hyperhomocysteine (HHcy)-inducing diet (7 females and 9 males).

Body fat, lean tissue content, and bone mineral density were assessed in mice subjected to either the control or HHcy-inducing diets for 15 weeks, using the non-invasive dual-energy X-ray absorptiometry (DXA) imaging methodology [38,39]. Imaging was performed using the iNSiGHT VET DXA system (Osteosys, Seoul, Republic of Korea), available at the NYU Langone’s Preclinical Imaging Laboratory. After instrument calibration with a standard phantom block, mice—under isofluorane anesthesia—were individually placed on the DXA scanner in prone position, with the limbs and tail stretched away from the body, and scanned from the neck down excluding the tail.

### 2.3. Tissue Collection and Processing

At the end of the study, with animals under isoflurane-induced deep anesthesia, blood was collected by cardiac puncture using sodium citrate as an anticoagulant. Following centrifugation at 13,000 rpm for 20 min, plasma samples were collected and stored at −80 °C until processing. Icteric plasma samples were excluded from the analysis. Brains were harvested and homogenized in ice-cold phosphate-buffered saline (PBS) at a ratio of 1 mL of PBS per 200 mg of tissue with the aid of a Dounce Homogenizer (Sigma-Aldrich, Burlington, MA, USA). Following centrifugation in a Beckman Optima XL-100K ultracentrifuge at 30,000 rpm (1 h at 4 °C), supernatants were collected, and stored at −80 °C until further analysis. Protein concentrations were assessed using bicinchoninic acid (BCA) protein assay (Thermo Fisher Scientific, Waltham, MA, USA).

### 2.4. Quantification of Homocysteine, Methionine, and Cysteine by LC-MS/MS Analysis

Mouse plasma samples (10 µL) were spiked with 10 µL of deuterated internal standard solution containing homocysteine-d4, methionine-d3, and cysteine-d3 (each at 35.92 µmol/L) and treated with DTT (3 µL, 500 mmol/L solution) to allow conversion of all the various bound and oxidized forms of the Hcy into its single, free, reduced form, as previously described [40,41,42,43,44]. This was followed by protein precipitation through the addition of 20 µL of 0.1% formic acid/0.05% TFA in acetonitrile, in agreement with previously described protocols [41,42,43,44], and subsequent centrifugation at 13,000 rpm for 15 min at 4 °C. The supernatants were transferred to LC-MS vials and analyzed using a Thermo Fisher Scientific Vanquish UPLC system coupled to a Q Exactive HF-X Orbitrap mass spectrometer (Thermo Fisher Scientific), operated in positive electrospray ionization (ESI) and parallel reaction monitoring (PRM) mode. Chromatographic separation was performed with an Acclaim^TM^ 120 C18 column (4.6 × 100 mm, 5 µm; Thermo Fisher Scientific) using a 10 min linear gradient and a 1 µL injection volume.

Calibration standards were prepared by spiking known concentrations of homocysteine, methionine, and cysteine in 0.1% FA to achieve final concentrations ranging from 1 to 200 µmol/L. Aqueous medium was used as a surrogate calibration approach to counterbalance the low-end background interference observed when using serum-base calibration which adversely affected the linearity of standard curves as the analytes are endogenous plasma components (shown for Hcy in Supplementary Materials S1). This approach enabled exploratory quantitation in limited-volume mouse plasma samples, which—based on the magnitude of the changes observed between the animals receiving either normal or special diets—fits the purpose of the current work. Quality control (QC) samples at low (8 µmol/L), mid (40 µmol/L), and high (120 µmol/L) concentrations were prepared independently and processed in parallel with study samples and calibration standards.

### 2.5. Assessment of Oxidative Stress in Brain Homogenates

#### 2.5.1. Glutathione Quantitation

Glutathione content in murine brain homogenates was assessed using the Glutathione Fluorescent Detection Kit (Fisher Scientific/Invitrogen), which allows quantitative determination of both glutathione (GSH) and its oxidized form (GSSG). Briefly, in accordance with the manufacturer’s protocol, 50 µL of either the homogenate samples (at 1:500 dilution) or the standard solutions were incubated, for 15 min at room temperature, with the non-fluorescent detection reagent which, upon binding to the free thiol group on GSH, yields a highly fluorescent product. Signal intensity was evaluated at 510 nm with excitation at 390 nm using a BioTek Synergy Neo2 Hybrid Multimode Reader (Agilent, Santa Clara, CA, USA). After free GSH evaluation and subsequent addition of a reaction mixture that converts GSSG into free GSH, the final fluorescence signal represented the total glutathione content, the sum of free GSH and GSSG.

#### 2.5.2. Assessment of Glutathione-S-Transferase (GST) Activity

GST enzymes catalyze the conjugation of GSH to a wide range of toxic and electrophilic compounds playing an important role in detoxification as well as antioxidants and modulators of oxidative stress [45,46]. Changes in the GST activity in mouse brain homogenates were assessed using the GST Assay Kit (Sigma) which evaluates the ability of the enzyme to catalyze the conjugation of GSH with the electrophilic substrate 1-chloro-2,4-dinitrobenzene, resulting in a product that can be evaluated spectrophotometrically over time. Following the manufacturer’s protocol, samples (4 µL of a 1:10 dilution in sample buffer) were combined with the substrate solution and absorbance was evaluated at 340 nm up to 5 min in a BioTek Synergy Neo2 Hybrid Multimode Reader. GST activity was expressed as (µmol/mL/min) for each timepoint.

#### 2.5.3. Determination of Lipid Peroxidation

Lipid peroxidation levels in mouse brain homogenates were assessed through the quantitation of Malondialdehyde (MDA), a commonly used biomarker of oxidative stress, using an MDA Assay Kit (Thermo Fisher/Invitrogen) in accordance with the manufacturer’s protocol. The assay is based on the reaction of MDA within the samples with Thiobarbituric Acid (TBA)—taking place in acidic conditions, at 100 °C—to generate the MDA-TBA adduct that is subsequently evaluated spectrophotometrically. Briefly, 20 µL of undiluted samples and standards were mixed, in accordance with the manufacturer’s protocol, with clarificant—which removes interfering substances, including proteins—together with an acid reagent, and the chromogenic agent TBA. This was followed by an incubation at 100 °C for 40 min, followed by a 10 min centrifugation at 10,000× *g*, and subsequent absorbance evaluation at 532 nm using a BioTek Synergy Neo2 Hybrid Multimode Reader (Agilent, Santa Clara, CA). MDA levels for each sample were calculated via interpolation with a standard curve, normalized to protein concentration assessed via BCA protein assay, and expressed in µmol/mg.

### 2.6. Statistical Analysis

Statistical comparisons were performed by unpaired *t*-test with Welch’s correction or 2-way ANOVA with Šídák’s multiple comparisons test using GraphPad Prism 10. Outliers were identified and excluded from the dataset via the ROUT method (Q = 10%). Regression and correlation analyses, and calculations of Spearman rank and two-sided Pearson correlation coefficients were performed using R software version 4.6.1 (https://www.r-project.org). In all cases, significance was set at *p* < 0.05.

## 3. Results

### 3.1. Effect of HHcy-Inducing Diet on Weight and Body Fat Content

**Figure 2:**
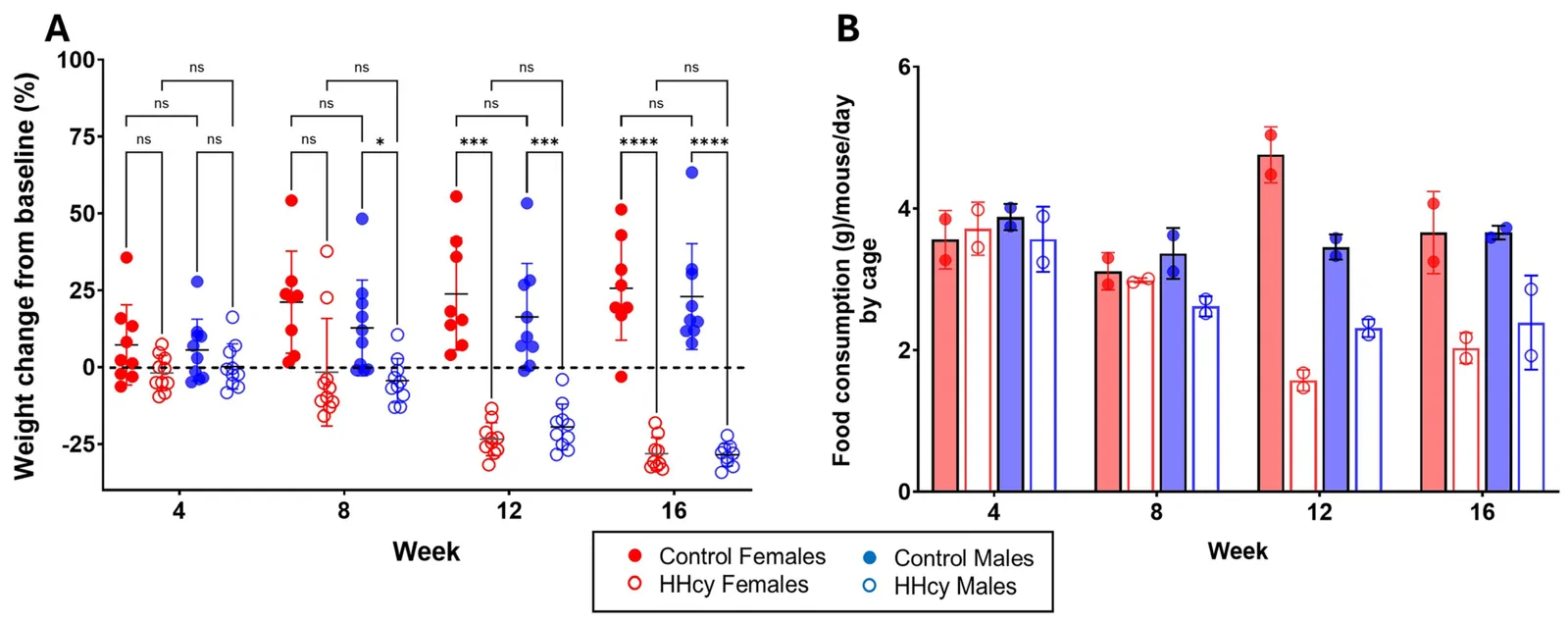
Body weight and food consumption in control and HHcy mice. (**A**) Weight changes expressed in % compared to baseline values at the beginning of the feeding experiment in controls and HHcy cohorts along the 16-week study. Data represents the mean ± SD. In all cases, ns indicates non-significant, * *p* < 0.05, *** *p* < 0.001, and **** *p* < 0.0001. (**B**) Food consumption in controls and HHcy cohorts during the 16-week study shown as an average value calculated by cage.

At the beginning of the diet study, which started when mice reached 6 months of age, their weight ranged between 21.5 and 27.4 g in females and 24.7 and 33.3 g in males, consistent with previously reported values in the C57BL/6 strain [47]. The mice on the control diet—both males and females—continued to gain weight over the course of the study with the % increase from baseline weight values reaching statistical significance starting at 12 weeks (*p* < 0.01 for both male and female cohorts). In contrast, mice fed a HHcy-inducing diet—consistent with previous reports [48,49]—lost weight from baseline values starting at 8 weeks, in the case of males, and more significantly after 12 weeks, in both males and females (Figure 2A) with no significant difference in the % of weight loss between genders.

Food consumption at the beginning of the diet experiment averaged between 3.3 and 3.9 g/mouse/day for both genders in control and HHcy groups, in agreement with previously reported values [47]. Consistent with previous work that indicated a decrease in food intake in mice receiving a comparable HHcy-inducing diet [49], a similar trend was observed in our case in animals of both genders after 12 and 16 weeks of feeding (Figure 2B). Since our experimental protocol evaluated food consumption by cage and therefore led to only two datapoints per group, statistical significance could not be accurately calculated. Whether the weight loss observed in animals receiving the special diet implicates additional more complex mechanisms than mere changes in appetite remains to be determined. Indeed, the association between elevated Hcy levels and weight loss may reflect metabolomic changes and disruptions in energy metabolism as reported in different rodent models than the one used in the current study [50,51].

**Figure 3:**
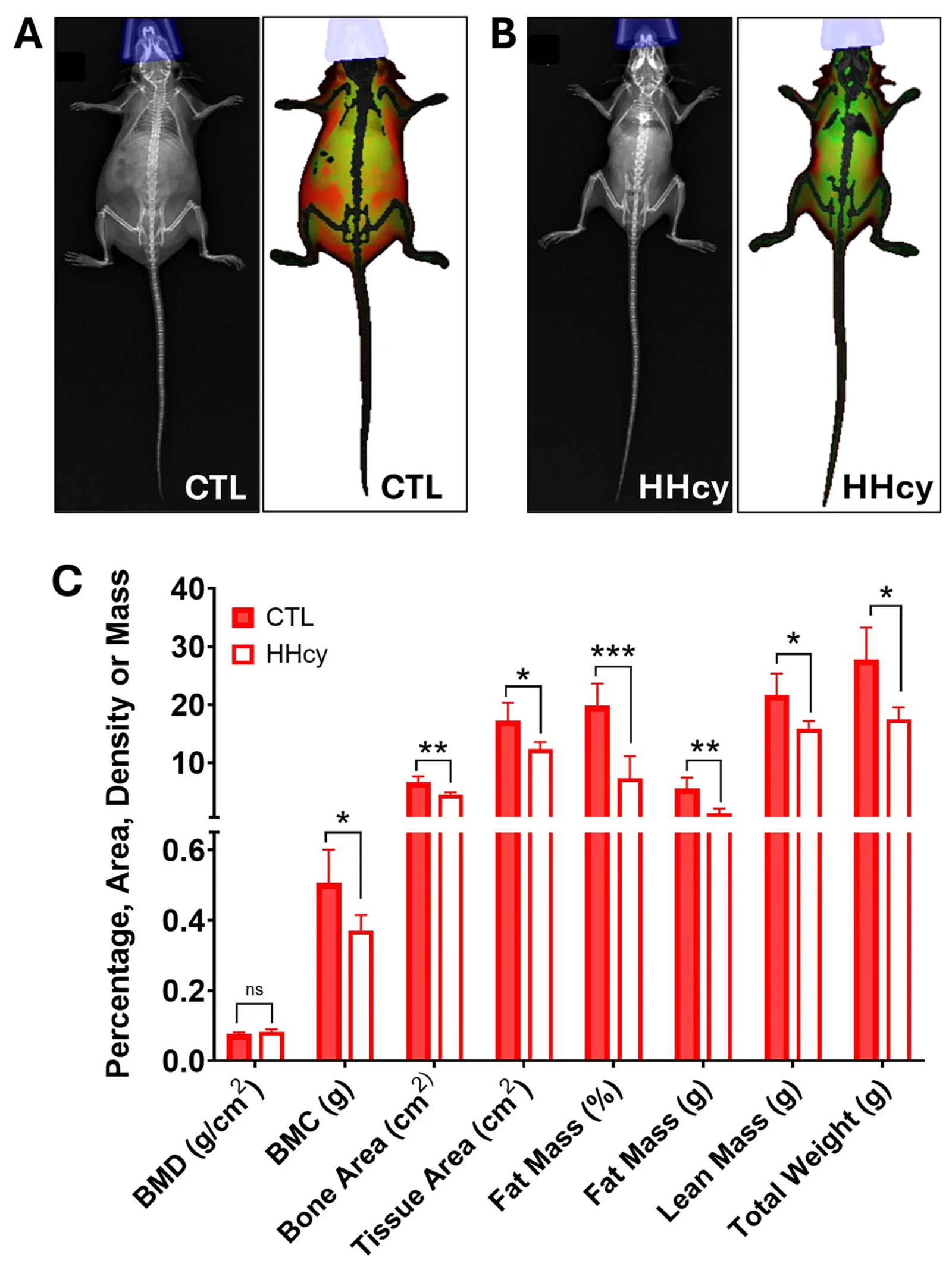
Dual-energy X-ray absorptiometry. (**A**) Representative images of skeleton and tissue composition from female mice fed a control diet for 15 weeks acquired using the iNSiGHT VET DXA system. (**B**) Representative DXA images from mice subjected to HHcy-inducing diet for 15 weeks. In both (**A**,**B**), left panels illustrate standard X-ray attenuation images providing anatomical reference and detailed images of the bone structures and internal organs; right panels indicate lean and fat masses depicted as green and red signals, respectively. (**C**) Bone and tissue composition parameters in control and HHcy cohorts (five and seven animals, respectively) calculated automatically by the DXA system integrated software. Data represents mean ± SD. In all cases, ns indicates non-significant, * *p* < 0.05, ** *p* < 0.01, and *** *p* < 0.001.

Additional imaging studies were performed to provide more detailed assessment of the effect of HHcy-inducing diets in C57BL/6 mice. Based on the similar weight loss observed between both genders at the terminal stages of the of feeding experiment (Figure 2A), DXA analysis was performed in two sets of female mice that had received either the control or the HHcy-inducing diets for 15 weeks to evaluate bone mineralization as well as changes in the fat and lean tissue composition. As illustrated in the images displayed in Figure 3A,B (right panels), the HHcy diet caused noticeable changes in the fat and lean tissue content, depicted as red and green signals, respectively. A quantitative assessment of DXA data (Figure 3C) demonstrated that although the 15-week diet did not cause significant changes in bone mineral density (BMD) it correlated with statistically significant decreases in bone mineral content (BMC), as well as in fat and lean tissue content, consistent with the decrease in body weight illustrated in Figure 2A.

### 3.2. Simultaneous Quantitation of Total Plasma Homocysteine, Methionine, and Cysteine Levels in Mice Fed Control and HHcy-Inducing Diets Assessed by LC-MS/MS

**Figure 4:**
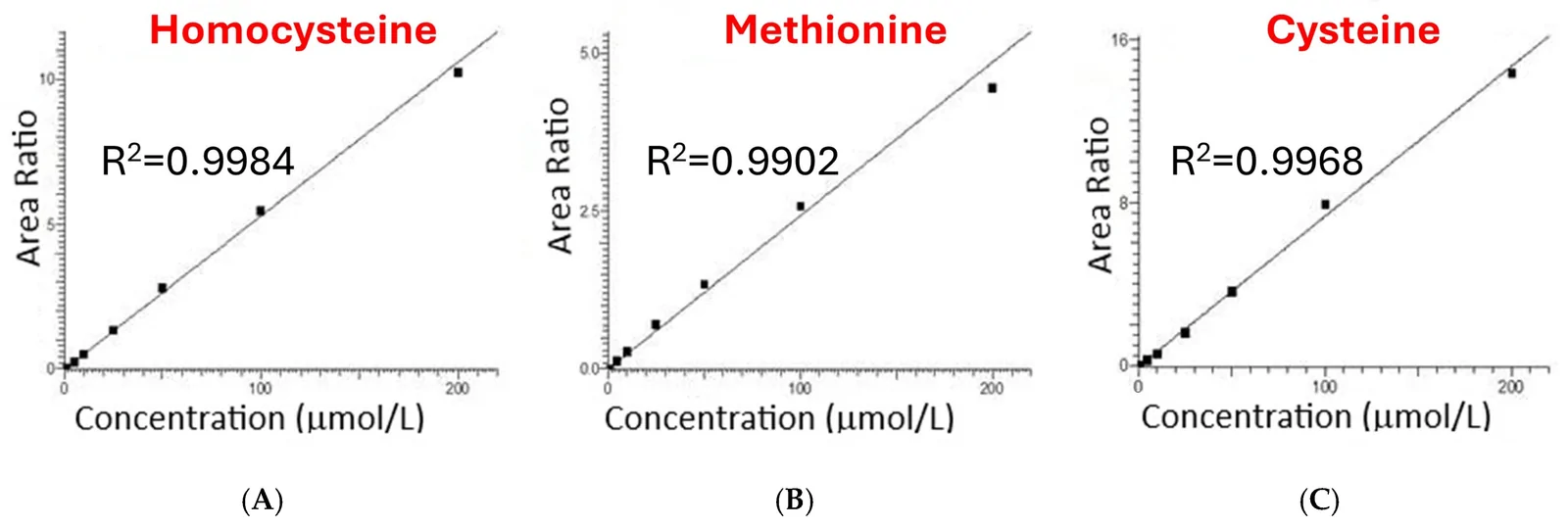
Calibration curves for the evaluation of homocysteine, methionine, and cysteine by LC-MS/MS. (**A**) Homocysteine calibration curve (R^2^ = 0.9984). (**B**) Methionine calibration curve (R^2^ = 0.9902). (**C**) Cysteine calibration curve (R^2^ = 0.9968). Area ratios were plotted against nominal concentrations and fitted using weighted (1/X) linear regression.

**Figure 5:**
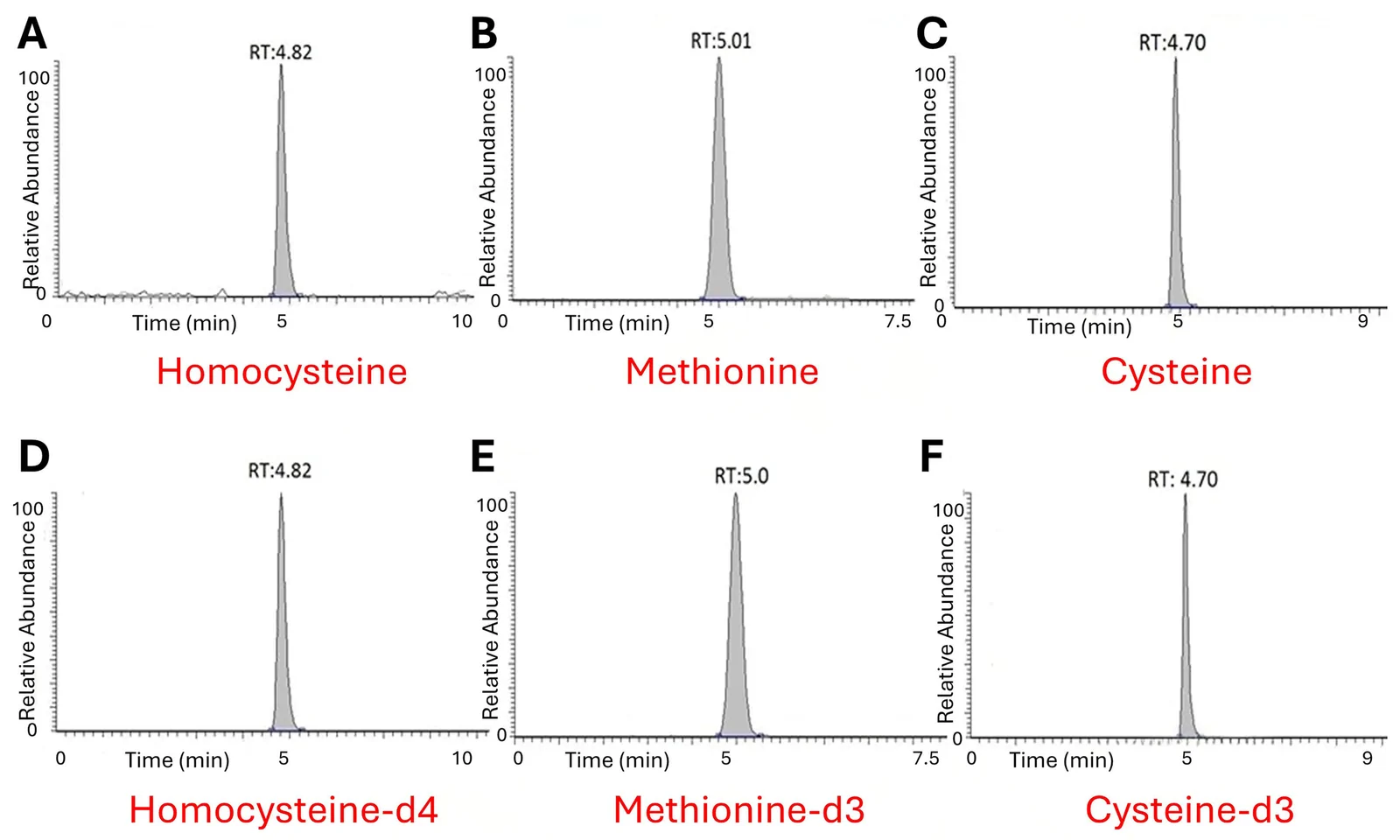
LC-MS/MS extracted ion chromatograms (XICs) of homocysteine, methionine, cysteine, and their corresponding isotopically labeled standards. Monitored transitions for (**A**) homocysteine (*m*/*z* 136.04 → 90.04); (**B**) methionine (*m*/*z* 150.06 → 104.05); (**C**) cysteine (*m*/*z* 122.03 → 76.02); (**D**) homocysteine-d_4_ (*m*/*z* 140.07 → 94.06); (**E**) methionine-d_3_ (*m*/*z* 153.08 → 107.07); and (**F**) cysteine-d_3_ (*m*/*z* 125.05 → 79.05).

Our customized LC-MS/MS procedure using 10 μL of mouse plasma demonstrated high linearity across the tested concentration ranges, with R^2^ values of 0.9984 for homocysteine (Figure 4A), 0.9902 for methionine (Figure 4B), and 0.9968 for cysteine (Figure 4C). The monitored transitions for each analyte were as follows: homocysteine (*m*/*z* 136.04 → 90.04), methionine (*m*/*z* 150.06 → 104.05), and cysteine (*m*/*z* 122.03 → 76.02) (Figure 5A–C for calibration standards and Supplementary Figure S1 for representative mouse plasma samples). Stable isotope-labeled internal standards, including homocysteine-d4 (*m*/*z* 140.07 → 94.06), methionine-d3 (*m*/*z* 153.08 → 107.07), and cysteine-d3 (*m*/*z* 125.05 → 79.05), were used to improve analytical consistency and partially account for variability during sample preparation and ionization (Figure 5D–F). Limits of detection (LOD) and limits of quantification (LOQ) were determined for each analyte, with homocysteine showing an LOD of 0.81 µmol/L and LOQ of 2.45 µmol/L, methionine with an LOD of 2.77 µmol/L and LOQ of 8.40 µmol/L, and cysteine with an LOD of 2.19 µmol/L and LOQ of 6.63 µmol/L (Table 1). The method’s precision was evaluated through intraday and interday validation, with coefficient of variation (%RSD) values ranging from 0.93% to 9.10% for intraday precision and 3.43% to 13.90% for interday precision across all quality control (QC) levels (Table 2 and Table 3). Intraday and interday validation data for individual replicates by QC level are shown in Supplementary Materials S2 and S3, respectively. Most QC measurements were within conventional ± 15% accuracy and ≤15% precision thresholds. Nevertheless, some values exceeded this criterion, including Met at the low QC level for interday precision and some high-QC accuracy values. These deviations indicate that—although the workflow constitutes a useful fit-for-purpose approach for exploratory preclinical profiling—additional work would be required to fully authenticate the methodology for clinical use. It is noteworthy to mention that—although full validation is pending—based on the magnitude of the amino acid level changes observed between the animals receiving either normal or special diets, the LC/MS-MS workflow constitutes a useful approach for our studies.

**Figure 6:**
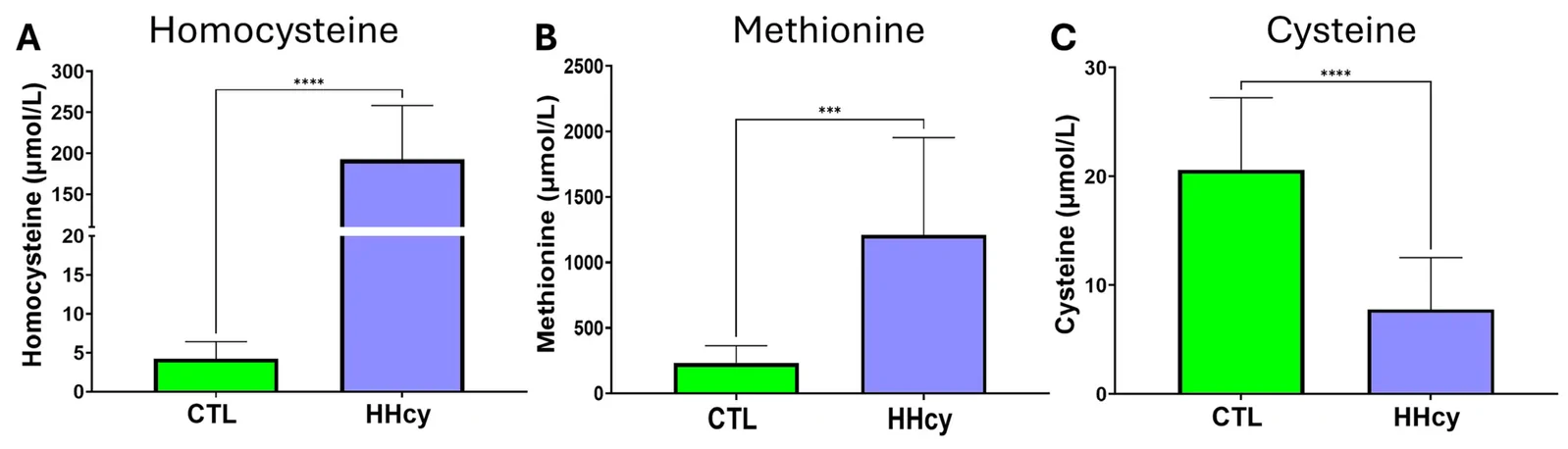
Plasma levels of total homocysteine, methionine, and cysteine in mice fed control and HHcy-inducing diets. (**A**) Hcy circulating levels averaged 4.2 ± 2.2 µmol/L in the control cohort and 192 ± 66 µmol/L in the HHcy mice. (**B**) Plasma Met increased to a mean value of 1209 ± 745 µmol/L in mice receiving the HHcy diet in comparison to the mean concentration of 234 ± 131 µmol/L exhibited by control-diet mice. (**C**) Plasma Cys mean values decreased to 7.8 ± 4.8 µmol/L in HHcy diet-fed mice compared to the mean concentration of 20.6 ± 6.6 µmol/L shown by mice subjected to the control diet. Data represents mean ± SD. In all cases, *** indicates *p* < 0.001 and **** *p* < 0.0001.

**Figure 7:**
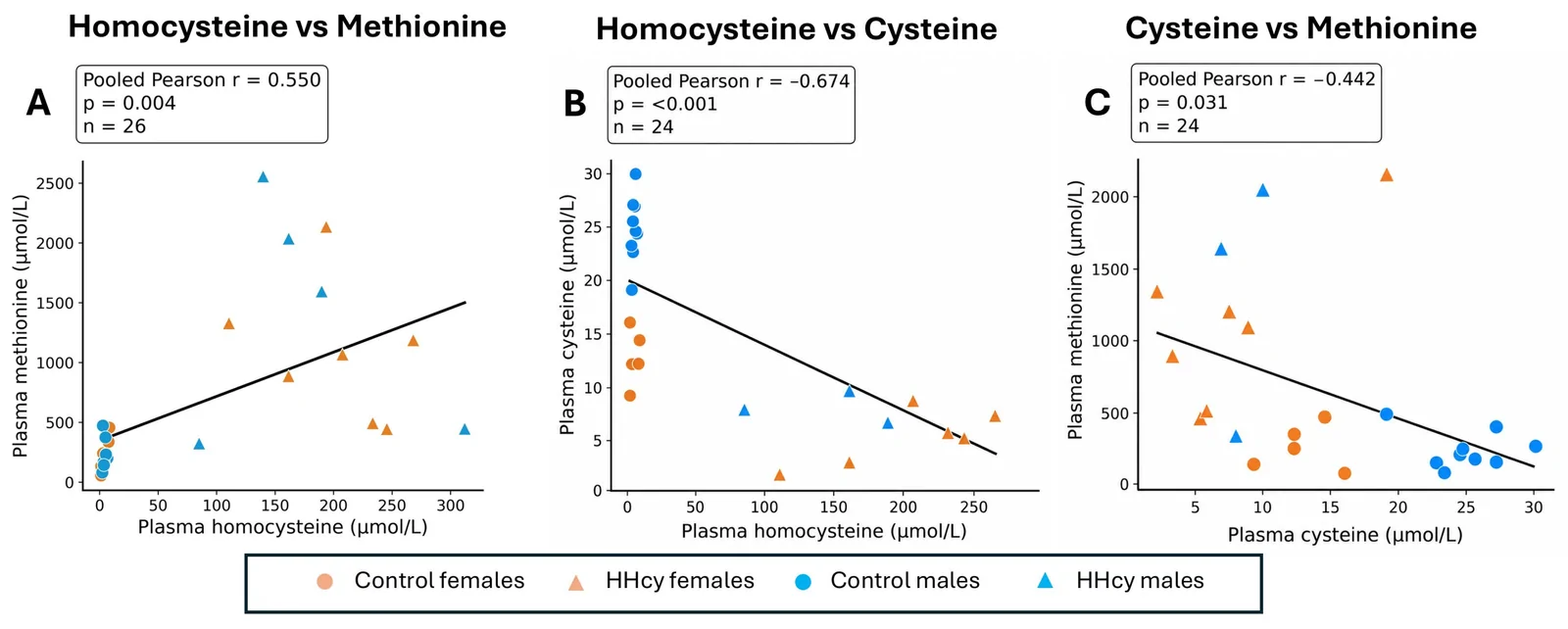
Plasma amino acid correlations. Pearson correlation analyses performed using R software (https://www.r-project.org) demonstrated the positive correlation between Hcy and Met levels (**A**) as well as the inverse correlation of Cys with Hcy and Met concentrations (**B**,**C**), respectively. In all cases *p* indicates unadjusted *p*-values.

Previous studies conducted in C57BL/6 mice fed HHcy-inducing diets reported the induction of moderate HHcy after 8, 12 or 14 weeks without describing changes in Met or Cys levels [15,17,52,53]. In our study, mice of both genders fed a control diet showed Hcy levels below 12 µmol/L (mean ± SD = 4.2 ± 2.2 µmol/L). In contrast, the majority of the mice subjected to the special diet for 16 weeks developed severe HHcy, as defined by values higher than 100 µmol/L (mean ± SD = 192 ± 66 µmol/L), as shown in Figure 6A and Table 4. The only exception was one mouse that only developed moderate HHcy (85 µmol/L). Whether this lower Hcy level is related to lower food ingestion remains to determined, as in our experimental design food consumption was evaluated per cage and not for individual mice. One additional animal that developed mild HHcy (26.5 µmol/L) but showed signs of poor health was excluded from the studies. Our studies also showed a statistically significant increase in Met (mean ± SD = 1209 ± 745 µmol/L) and a decrease in Cys (mean ± SD = 7.8 ± 4.8 µmol/L) plasma levels in HHcy mice compared to animals receiving control diets, which showed Met and Cys values of 234 ±130 µmol/L and 20.6 ± 6.6 µmol/L, respectively (Figure 6B,C and Table 4). It should be noted that, of the amino-acid concentration changes induced by the diet, the marked elevation in plasma Hcy and Met in the HHcy group is particularly relevant as the amino acid levels detected are well above the LOQ. Caution should be taken in reference to the accuracy of the low Cys concentrations observed in the HHcy mouse cohort, as the mean values in the animals receiving the special diet dropped to a mean of 7.8 µmol/L, levels close to the LOQ of 6.63 µmol/L (Table 1). Although in the absence of formal matrix effect evaluation these low Cys values may reflect limited precision, the effect of the special diet on the plasma concentrations of the amino acid is noteworthy. For the three analytes, data from both genders were combined as no significant differences were found between females and males. All data for individual replicates are provided in Supplementary Materials S4. Pearson correlation analyses (Figure 7) showed a statistically significant positive correlation between Hcy and Met plasma concentrations (r = 0.550, *p* = 0.004) and indicated a significant inverse correlation of Cys levels with Hcy (r = −0.674, *p* < 0.001) and Met (r = −0.442, *p* = 0.031) concentrations.

It should be mentioned that, although significant statistical differences were detected between the normal and HHcy-inducing diet cohorts for the three analytes tested by LC-MS/MS—Hcy, Met, and Cys, a high standard deviation was observed in all cases. This likely reflects, at least in part, the limited number of animals available for analysis at the end of the 16-week feeding experiment, particularly in animals receiving the special diet, which exhibited higher mortality than those fed the normal chow, as has been previously reported in mice [49] and in humans with extreme HHcy [54]. Future studies with a larger starting sample size may provide an estimate of the population parameters with reduced variability. The substantial inter-animal variability in plasma amino-acid concentrations could additionally indicate the effect of potential co-variates modifying the metabolic response to the diet as gender, animal weight, and/or food consumption. Regression analyses indicated that, in animals with complete endpoint data, the observed heterogeneity in plasma Hcy, Met, and Cys concentrations within the HHcy cohort is not significantly predicted by sex or terminal body weight, as illustrated by the statistical parameters shown in Table 5. Food consumption could not be incorporated as an individual-level predictor since, as indicated above, intake was recorded per cage rather than per individual mouse, resulting in only two datapoints per group.

### 3.3. Analysis of Oxidative Stress Markers in Brain Homogenates of Mice Fed Control and HHcy-Inducing Diets

**Figure 8:**
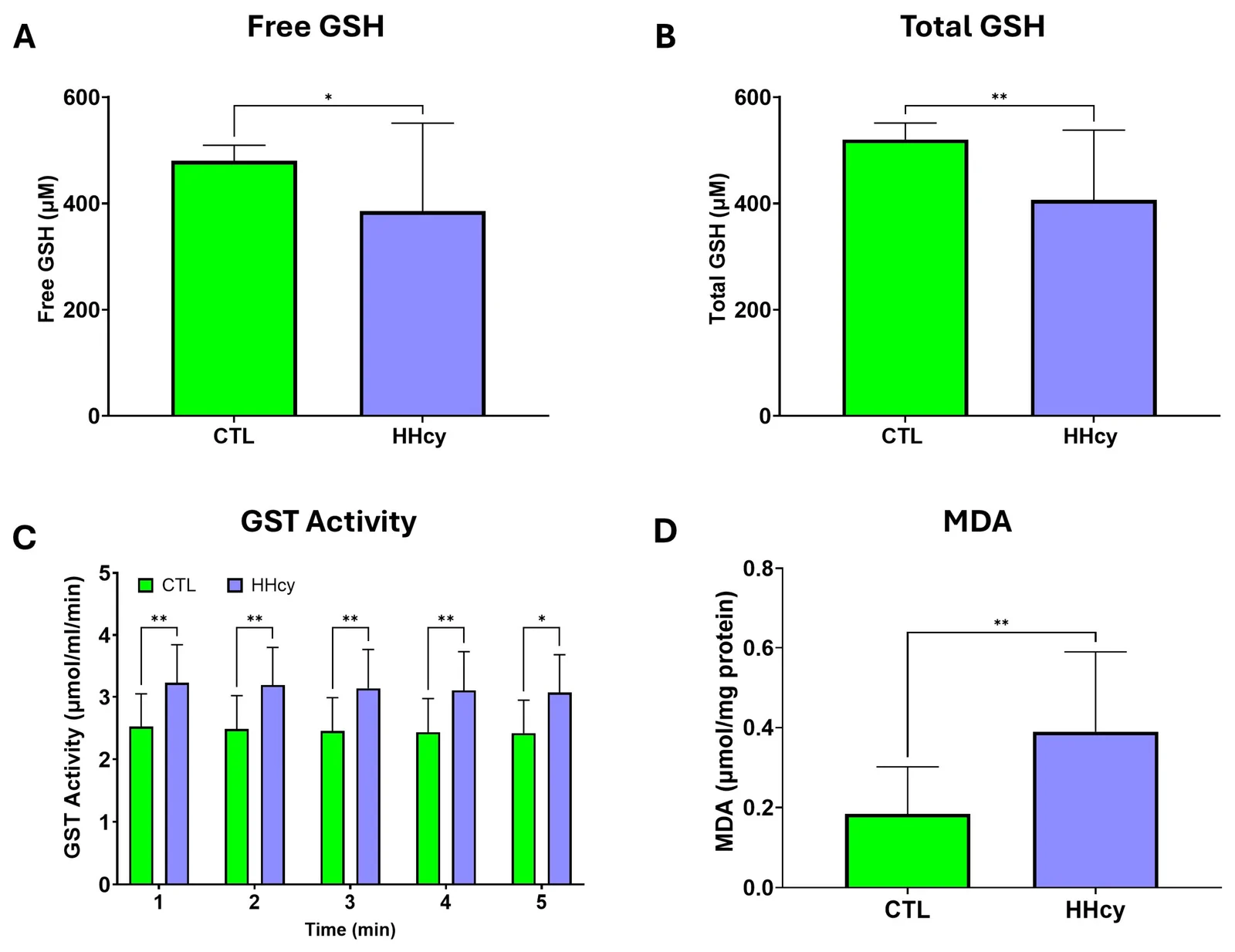
Oxidative stress markers in brain homogenates of control and HHcy mice. A significant reduction in free (**A**) and total (**B**) glutathione levels, assessed using the Glutathione Fluorescent Detection Kit, were observed in the HHcy cohort. (**C**) Enzymatic activity of the antioxidant enzyme Glutathione S-transferase (GST), evaluated for up to 5 min, was significantly increased in HHcy mice compared to control animals. (**D**) Lipid peroxidation assessed through the quantitation of MDA, was significantly increased in the HHcy cohort. Data represents the mean ± SD. In all cases, * indicates *p* < 0.05 and ** *p* < 0.01.

Cysteine is the precursor of glutathione, one of the most important antioxidant molecules protecting cells from the detrimental effect of ROS [4,5]. The current research investigated whether the decrease in Cys levels resulting from the HHcy-inducing diet affected glutathione levels and thereby impacted the antioxidant defense systems. Brain homogenates from mice fed the HHcy diet showed decreased levels of both total and free glutathione (GSH) compared to control-diet mice (Figure 8A,B). A concomitant increase in the activity of GST, a crucial antioxidant enzyme protecting cells from the effect of ROS (Figure 8C), was also observed in the HHcy cohort. As an assessment of the induction of oxidative stress in brain tissue homogenates, our work evaluated lipid peroxidation through the detection of MDA, a reactive aldehyde resulting from the peroxidation of polyunsaturated fatty acids. Consistent with the reduced levels of GSH shown above, MDA concentrations were significantly increased in the HHcy cohort, consistent with the presence of oxidative stress conditions (Figure 8D). Based on the absence of significant differences between females and males, Figure 8A–D illustrate the combination of data from both sexes.

**Figure 9:**
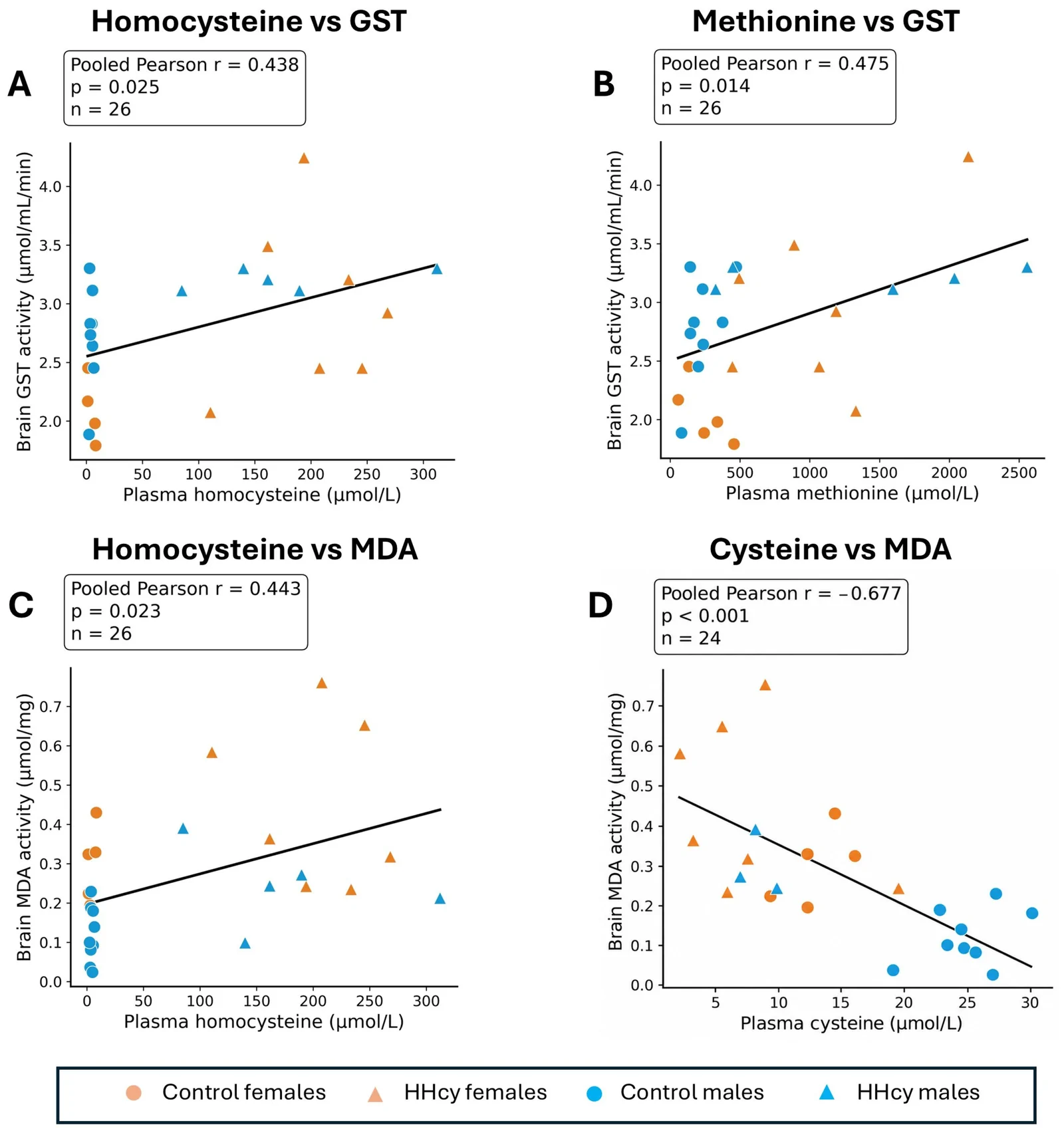
Correlation between plasma amino acid levels and oxidative stress markers in brain homogenates. Pearson analyses performed using R software show positive correlation between Hcy and GST activity (**A**) as well as between Met levels and GST (**B**). Hcy levels correlated positively with the oxidative stress marker MDA (**C**) while Cys exhibited a statistically significant inverse association with MDA (**D**). In all cases *p* indicates unadjusted *p*-values.

Pearson correlation analyses showed that, across the complete dataset, higher plasma Hcy and Met concentrations were associated with higher GST activity (Figure 9A,B). Further reinforcing the association between altered sulfur-amino-acid metabolism and oxidative stress in brain tissue, Hcy correlated positively MDA levels (Figure 9C) and tended to associate with lower total GSH (r = −0.380, *p* = 0.05). In turn, Cys showed in the full cohort a strong inverse association with MDA (Figure 9D). The remaining pooled correlations were not statistically significant.

## 4. Discussion

HHcy is a condition characterized by elevated blood levels of Hcy, an amino acid positioned at a central intermediate point within Met and Cys metabolic pathways. As such, Hcy can either be methylated to generate Met through the B12-dependent enzyme MS or irreversibly converted into the Cys precursor cystathionine by CBS, a vitamin B6-dependent enzyme (Figure 1) [1,7]. Due to common deficiencies in B vitamins during aging, HHcy is remarkably common among the elderly population. Along this line, elevated circulating Hcy concentrations have been associated with the development of cerebrovascular diseases and other age-associated conditions, among them neurodegenerative disorders such as Alzheimer’s and Parkinson’s diseases, as well as different eye pathologies including age-related macular degeneration and diabetic retinopathy [55,56,57,58,59,60,61]. In many of these HHcy-induced neurodegenerative and eye disorders, disruption of the cerebral and retinal blood barrier function with subsequent triggering of inflammation-related pathways are important contributors to the underlying pathogenesis [62,63].

Various analytical techniques with varying sensitivity and sample volume requirements have been employed to quantify total Hcy along with the metabolically related amino acids Met and Cys, including immunoassays as well as HPLC- and LC-MS/MS-based methodologies. Immunoassays, although widely used in clinical settings for their high-throughput capabilities, can exhibit not only sample interferences when using complex biospecimens as plasma and other biological materials but also limited specificity, particularly resulting from cross-reactivity with structurally related metabolites, as is the case with Cys interference in the quantification of Hcy [30]. In contrast, LC-MS/MS provides superior specificity, accuracy, and multiplexing capabilities, making this methodology the preferred approach for research settings and biomarker evaluation. Our LC-MS/MS assay for the simultaneous quantitation of Hcy, Met, and Cys was adapted for a 10 µL low-volume murine plasma sample, addressing the limitations posed by sample availability in small-animal studies. Unlike many published methods that involve derivatization or solid-phase extraction, our approach employs only DTT-mediated disulfide reduction followed by protein precipitation, enabling rapid and reproducible sample preparation. While the limits of detection and quantification for Met and Cys are slightly higher than those achieved by triple quadrupole-based Multiple Reaction Monitoring (MRM) methods [64,65], the current methodology provides analytical performance sufficient for preclinical research in small animals.

LC–MS/MS quantifications in complex biological samples are often complicated by the presence of endogenous analytes and the difficulty in obtaining analyte-free biological matrices to create reference samples. A common strategy to overcome this challenge is the use of surrogate matrices which, although they may not entirely replicate all matrix effects of plasma, are well established for improving assay linearity and accuracy particularly in cases in which an analyte-free biological matrix is unavailable or of impractical use for standard curve generation [32,66,67,68]. In the current work, accurate calibration curves were generated using a 0.1% FA solution in water (Figure 4). This approach minimized endogenous background interference and enabled precise detection of low-abundance analytes (Figure 5). The current LC-MS/MS methodology was adapted from previous work encompassing detailed optimization and recovery assessment protocols for the quantitation of homocysteine [36,43]. We acknowledge as a limitation of our current studies the absence of both post-extraction spiking experiments with stable isotope-labeled Hcy, Met, and Cys as internal standards to fully assess matrix effect and analyte recovery evaluation. These experiments are warranted to provide definitive indication of whether—and to what extent—the use of plasma as a matrix alters ion signal and, therefore, influences the final quantitative outcome of the three analytes [69,70]. However, it should be mentioned that heavy isotope-labeled internal standards were included in each assay to control for potential matrix effects. Despite the limitations of the study and based on the magnitude of the amino-acid concentration changes observed in our experimental design, the LC/MS-MS workflow used herein constitutes a useful approach to answer the biological questions posed by our studies.

Modifications to rodent diets that typically either provide excessive Met or are deficient in vitamin B have been used to induce HHcy [17,52,71]. Despite both diet modifications resulting in increased Hcy levels, these two approaches differ in the mechanism by which they increase the concentration of the amino acid. While high Met intake promotes Hcy and Cys synthesis without limiting the production of Met, vitamin B deficiency inhibits the conversion of Hcy to Met and Cys, leading to increased Hcy together with Met depletion, as illustrated in Figure 1B [14]. Dietary strategies separately triggering these pathways have been shown to induce moderate HHcy, with levels lower than 30 µmol/L in both C57BL/6 and APP transgenic mice [17,52,72]. In contrast, dietary combinations consisting in elevated Met intake together with deficiency of B vitamins translated into severe HHcy, with reported mean values ranging from 140 to 169 µmol/L in both C57BL/6 and APP transgenic mice, depending on the duration of the special diet [48,49,72]. In our case, in which C57BL/6 mice were fed a high-Met and no-vitamin-B diet for 16 weeks, longer than in previous reports in which the animals received the special diet for up to 12 or 14 weeks [48,49], mice developed Hcy levels with a mean value of 192 ± 66 µmol/L (Figure 6A), higher than in work employing shorter treatments. In contrast, C57BL/6 mice, both males and females, receiving a control standard diet showed normal Hcy concentrations with a mean value of 4.2 ± 2.2 µmol/L, in agreement with previous studies conducted on the same strain [11,13,15,52].

The current work also demonstrates that HHcy mice, in comparison to animals receiving the normal chow, exhibited a significant increase in plasma Met—an anticipated finding consistent with the elevated intake of the amino acid—with concentrations reaching a mean value of 1209 ± 745 µmol/L (Figure 6B). It should be mentioned that, although a positive correlation between plasma Hcy concentration and Met levels present in the HHcy-inducing chow is well established [13,73], changes in the circulating Met concentration in the animals receiving the special diet have not been investigated with the same frequency. Mice fed the HHcy special diet also exhibited a decrease in plasma Cys with a mean concentration of 9.2 ± 6.5 µmol/L, a statistically significant difference from animals with the normal diet (Figure 6C), a particularly relevant—although expected—finding taking into consideration the role of this amino acid in the regulation of oxidative stress homeostasis.

**Figure 10:**
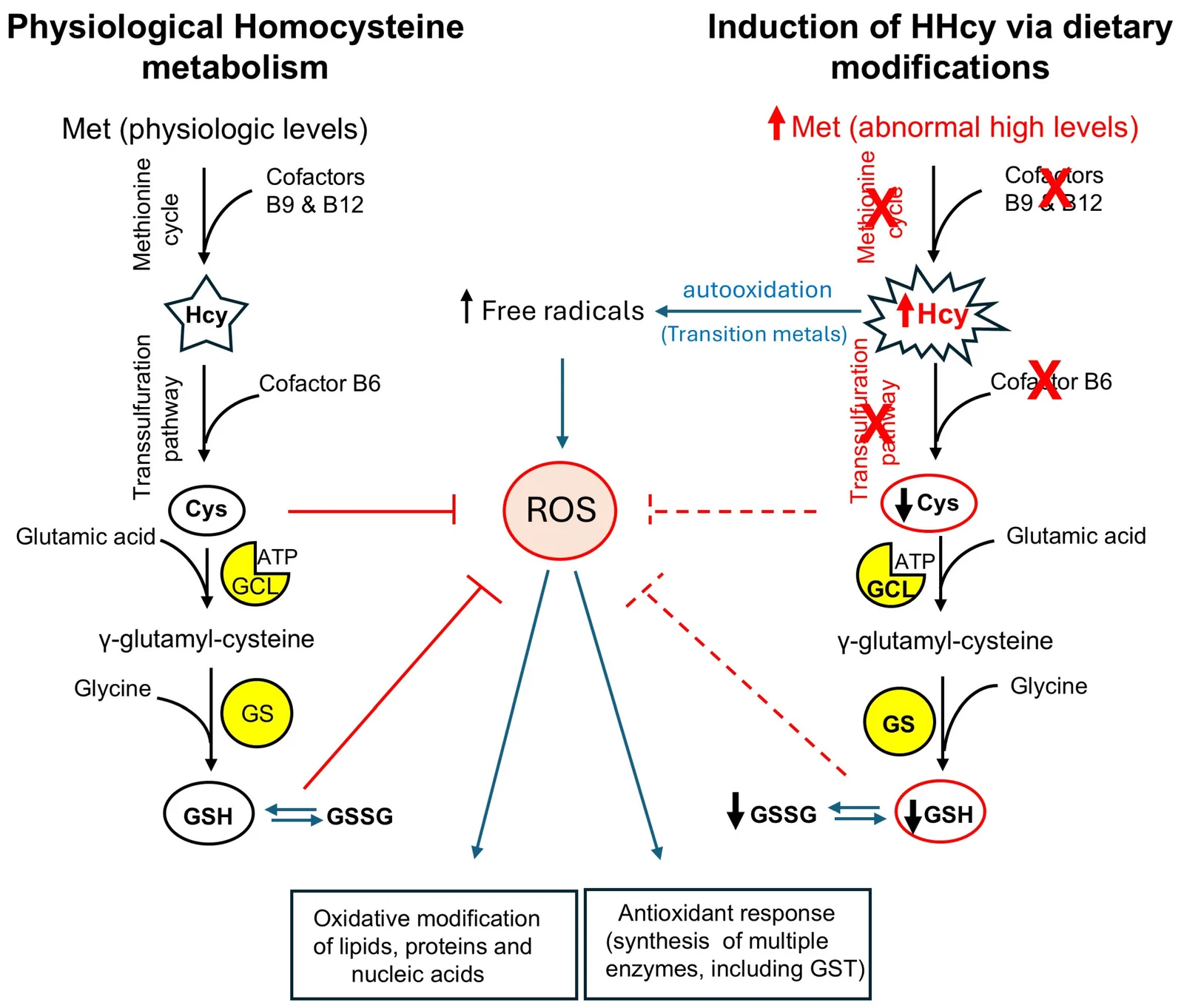
Schematic diagram linking methionine metabolism with HHcy and disbalance in ROS homeostasis. The figure illustrates how the dietary-induced changes in the physiological metabolism of Hcy that lead to elevated levels of the amino acid alter redox balance through both Hcy auto-oxidation and by decreasing the production of Cys, itself a modulator of the antioxidant response. The reduced Cys levels also translate in alterations of both reduced and oxidized forms of glutathione, the major intracellular non-enzymatic antioxidant. In turn, the increase in ROS generation causes undesirable oxidative modifications of lipids, proteins and nucleic acids and triggers the enhanced expression of antioxidant enzymes, including GST evaluated in the current work. GCL indicates glutamate-cysteine ligase; GS, glutathione synthetase; GSH, reduced glutathione; GSSG, oxidized glutathione; GST, glutathione S-transferase.

Cys plays a key role in antioxidant defenses not only in its capacity as a free radical scavenger, but also as the precursor of glutathione, one of the most important antioxidant molecules involved in the cellular protection from the detrimental effect of free radicals and oxidative stress-mediated damage [3,4]. The formation of glutathione involves in the first step the reaction between glutamic acid and Cys to form γ-glutamyl-cysteine—a process catalyzed by the enzyme glutamate-cysteine ligase (GCL) in the presence of ATP—followed by the addition of glycine through the action of glutathione synthetase (GS) [3,5] (Figure 10). Hcy is intricately related to Cys metabolic pathways and the concomitant regulation of ROS homeostasis. Indeed, the enhanced production of free radicals and decreased antioxidant cellular responses have been attributed a crucial role in HHcy pathogenetic effects, which can extend from detrimental changes limited to the vasculature to the development of chronic conditions and multiorgan failure system [74,75]. The most studied effects of HHcy are those associated with changes in endothelial function, cerebrovascular alterations, impairment of BBB membrane permeability, and the development of intracerebral hemorrhages, all changes that have been linked, at least in part, to a disruption in ROS homeostasis and concomitant induction of oxidative stress [52,55,56,76,77].

The disbalance in ROS homeostasis is frequently evaluated in research settings through the assessment of oxidative stress markers, as lipid peroxidation, as well as by the estimation of different elements of the antioxidant defense mechanisms. Many of the studies assessing HHcy-mediated oxidative damage have centered on the use of plasma specimens with few reports employing aorta and heart tissues [78,79]. The studies evaluating oxidative stress in the brain, one of the most affected organs in HHcy, are limited and mostly reflect the use of intracerebral injections of Hcy to increase the amino acid levels [18,76,78,80,81,82,83]. The current work evaluates the brain oxidative damage in C57BL/6 mice with diet-induced severe HHcy through the assessment of MDA—a marker of lipid peroxide formation—as well as via the evaluation of the antioxidant response by estimation of GST activity together with the quantitation of total and free forms of glutathione. It should be mentioned that, while quantification of total glutathione evaluates all glutathione forms including reduced (GSH), oxidized (GSSG), and protein-bound species, the assessment of free glutathione specifically measures the unbound reduced and oxidized forms, estimating in this way the immediate antioxidant availability. The special diet provided for a 16-week period resulted in reduced levels of both total and free forms of glutathione in brain homogenates (Figure 8A,B), consistent with previous findings that used different approaches to induce HHcy [76,83,84].

The impairment of the redox balance in HHcy-fed mice was also highlighted in our studies by the demonstration of increased enzymatic activity of GST (Figure 8C), likely reflecting a compensatory effect to counterbalance the diet-induced pro-oxidant conditions. This enzyme belongs to a protein family composed of several isoforms located in different cellular populations and organs that act as crucial contributors in the regulation of oxidative stress, playing a pivotal role in the conjugation of the thiol group of glutathione to electrophilic and toxic compounds facilitating their inactivation and elimination [45,46,85]. To our knowledge, no studies have reported the enzymatic GST activity in mice receiving HHcy-inducing diets, although a positive correlation between specific GST isoform activity and elevated Hcy plasma levels has been reported in patients with chronic kidney disease [86,87].

Lipid molecules, as a consequence of their high content of unsaturated bonds in the fatty acid chains, are especially vulnerable to oxidative stress [88]. The attack of free radicals on the double bonds of these fatty acid molecules initiates a series of reactions that lead to the formation of stable endpoint molecules of various types including aldehydes, ketones, alkanes, carboxylic acids, and polymerization products [89]. Among these molecules, the toxic aldehyde MDA is one of the most studied markers of lipid peroxidation and its quantification is critical for the assessment of the extent of the oxidative damage. The data presented in the current work demonstrate that the dietary modifications, which caused severe HHcy, doubled MDA levels in brain homogenates in comparison to concentrations found in mice receiving the control chow (Figure 8D). These findings are consistent with previous reports demonstrating MDA elevation in brain and heart tissues following either intracerebral Hcy administration or different dietary modifications from those employed herein, including work that showed increased plasma MDA following Hcy incorporation into the daily water source, all approaches that resulted in either undisclosed or lower Hcy plasma concentration than in our studies [83,84,90,91,92].

Consistent with our findings and as detailed above, studies in different animal models subjected to genetic manipulations or receiving special dietary modifications have demonstrated the association of HHcy with oxidative stress [93,94]. In humans, a large body of evidence emphasizes the significant role of oxidative stress in Hcy-induced pathological conditions, including endothelial dysfunction, atherosclerosis, age-related eye diseases, and neurodegenerative disorders [95,96]. Although Hcy plasma concentrations exceeding 100 µmol/L—as in the current model—and even reaching up to 500 µmol/L in severe cases have also been reported in humans, these extreme elevations have almost always been associated with genetic disorders rather than with dietary deficiencies [97,98]. Nevertheless, it should be highlighted that the association of HHcy with the induction of oxidative stress as a mechanistic basis of disease pathogenesis has been reported not only in cases of severely increased values consistent with those observed in our experimental model [99], but also within the full spectrum of elevated Hcy levels. In this sense, moderate Hcy increments with mean values ranging within 15 to 25 µmol/L have been associated with oxidative stress markers in AD as well as in patients with various cardiovascular abnormalities [20,72,100,101,102,103].

The current work links the increased plasma concentrations of Hcy and the concomitant decreased levels of the metabolically related amino acid Cys to the induction of oxidative stress in brain tissue. Although our study does not evaluate the concentrations of the amino acids in brain homogenates, it could be speculated—based on previously published work—that the changes in the circulating amino acid levels are likely to be recapitulated in the brain. In this sense animals receiving comparable diets to the one in our experimental model showed increased plasma Hcy concentrations which correlated with elevated levels of the amino acid in brain homogenates [104,105]. Animal studies inducing HHcy via genetic depletion of CBS also recapitulated in brain homogenates the elevation in Hcy and the decrease in Cys concentrations observed in plasma in our diet-induced model [106]. Notably, the decreased brain levels of Cys present in the genetically modified model, associated—in turn—with a concomitant decrease in GSH [106], highlight the relevance of the metabolic changes to the induction of oxidative stress, as observed herein. Whether the correlation between the amino acid changes in plasma and the induction of oxidative stress markers in brain tissue described in the current work reflect intrinsic brain mechanisms or are associated at least to some extent with BBB leakage remains to be determined. Along this line, it is noteworthy that comparable diet-induced, as well as genetically modified, HHcy models have shown disruption of barrier permeability integrity, extracellular matrix remodeling, and changes in the expression of matrix metalloproteases coexisting with development of microhemorrhages [52,104,107].

Overall, our work links the dietary-induced severe HHcy in C57BL/6 mice with a decrease in Cys plasma levels and the downregulation of glutathione. These metabolic changes, in turn, are associated with a concomitant reduction in the antioxidant response in brain tissue, as evaluated through the compensatory increased activity of the antioxidant enzyme GST as well as an increase in MDA, an indicator of lipid peroxide formation (Figure 10).

## 5. Conclusions

HHcy, a remarkably common condition among the aging population, has been associated with the development of cardiovascular disorders, neurodegenerative conditions, and eye diseases frequently found in elderly individuals. Although the specific underlying mechanisms by which elevated Hcy contribute to the molecular pathogeneses of these disorders remain to be fully elucidated, mounting evidence indicates a prominent role for the amino acid mediated dysregulation of ROS homeostasis. The current work—through the induction of severe HHcy in wild-type C57BL/6 mice via dietary modifications—provides insight into the mechanisms by which Hcy elicits oxidative stress conditions. The studies, made possible through the development of a fit-for-purpose LC-MS/MS methodology for the concurrent quantification of multiple analytes in low-volume plasma specimens, demonstrated that the elevated levels of Hcy affected the metabolic pathways of the amino acid and reduced circulating Cys concentrations. In turn, the reduced Cys levels resulting from the dietary modifications translated into decreased concentrations of glutathione, a major regulator of the antioxidant defense responses. These metabolic alterations generated oxidative stress conditions as evidenced by a compensatory increase in the activity of the antioxidant enzyme GST and elevated levels of the lipid peroxidation marker MDA in brain homogenates. Overall, the current findings—through the use of an LC-MS/MS technique that shows increased specificity and accuracy compared to alternative immunoassays or HPLC-based methods while employing reduced volume samples—expands the translational potential of the simultaneous metabolite detection in small-animal preclinical studies. From a mechanistic point of view, the current work highlights the relevance of nutritional aspects in the pathogenesis of age-related disorders emphasizing the potential contribution of dietary modifications as combinatorial therapeutic strategies. Indeed, based on the complexity of the molecular mechanisms involved in these diseases, it is increasingly evident that future successful therapeutic modalities will likely require complex strategies targeting multiple pathways.

## Figures and Tables

**Table 1:** Limits of detection (LOD) and limits of quantification (LOQ) for homocysteine, methionine, and cysteine measured by LC-MS/MS.

Compound	LOD (µmol/L)	LOQ (µmol/L)
Homocysteine	0.81	2.45
Methionine	2.77	8.40
Cysteine	2.19	6.63

**Table 2:** Intraday performance of LC-MS/MS assay. Intraday precision, accuracy, and average measured concentrations for cysteine, methionine, and homocysteine (n = 10). Quality control (QC) samples at three concentration levels (QC1–QC3; target concentrations of 8, 40, and 120 µmol/L, respectively) were used to assess method performance. Precision is expressed as %RSD, and accuracy as % difference from target values.

**Intraday Average (n = 10)**			
QC Level	Cysteine (µmol/L)	Methionine (µmol/L)	Homocysteine (µmol/L)
QC 1	7.25	8.82	6.81
QC 2	38.66	37.67	33.74
QC 3	113.89	100.80	99.89
**Intraday Precision (n = 10)**			
QC Level	Cysteine (%RSD)	Methionine (%RSD)	Homocysteine (%RSD)
QC 1	1.99	1.33	1.09
QC 2	2.29	0.72	1.00
QC 3	9.10	0.93	1.51
**Intraday Accuracy**			
QC Level	Cysteine (%Diff)	Methionine (%Diff)	Homocysteine (%Diff)
QC 1	−9.33	10.31	−14.92
QC 2	−3.34	−5.82	−15.65
QC 3	−5.09	−16.00	−16.76

**Table 3:** Interday assay performance for LC-MS/MS quantification of cysteine, methionine, and homocysteine. Summary of measured concentrations, accuracy, and precision for three quality control (QC) levels (n = 10 per day) across three independent days. QC targets were 8, 40, and 120 µmol/L for QC1, QC2, and QC3, respectively. Accuracy is presented as a percentage difference from the target. Precision is expressed as %RSD.

**Daily Average (n = 10)**			
	***Cysteine***	***Methionine***	***Homocysteine***
	Day 1	Day 2	Day 3
Level	Mean	Mean	Mean
QC 1	7.25	8.82	6.81
QC 2	38.66	37.67	33.74
QC 3	113.89	100.80	99.89
**3-Day Average**			
	Conc (µmol/L)	Conc (µmol/L)	Conc (µmol/L)
QC 1	7.63	7.82	7.22
QC 2	36.69	35.71	35.22
QC 3	104.86	100.35	102.38
**3-Day Standard Deviation**			
	SD	SD	SD
QC 1	±1.06	±1.43	±0.58
QC 2	±2.60	±2.78	±2.09
QC 3	±7.83	±0.64	±3.51
**3-Day Accuracy**			
	% Diff	% Diff	% Diff
QC 1	−4.65	−2.31	−9.79
QC 2	−8.27	−10.73	−11.96
QC 3	−12.62	−16.38	−14.69
**3-Day Precision**			
	% RSD	% RSD	% RSD
QC 1	13.90	18.26	8.05
QC 2	7.09	7.79	5.93
QC 3	7.47	0.64	3.43

**Table 4:** Average plasma concentration (µmol/L) for homocysteine, methionine, and cysteine measured by LC-MS/MS in control and HHcy cohorts. Average of plasma concentrations (µmol/L) of homocysteine, methionine and cysteine with standard deviation measured by LC-MS/MS in control and HHcy mice. All compounds show a significant difference between control and HHcy diet as indicated by *p*-values.

Compound	Control Diet (µmol/L)	HHcy Diet (µmol/L)	*p*-Value
Homocysteine	4.25 ± 2.22	192.36 ± 65.82	<0.0001
Methionine	234.03 ± 130.75	1209.14 ± 745.06	0.0008
Cysteine	20.63 ± 6.58	7.77 ± 4.77	0.0003

**Table 5:** Separate Ordinary Least Square models for plasma Hcy, Met, and Cys using sex and terminal body weight as predictors. Regression analyses indicate that the observed heterogeneity in amino acid plasma levels does not relate to sex or terminal body weight in the available dataset within the HHcy group.

Outcome	n	Predictor	Estimate	SE	*p*-Value	R^2^
Hcy	12	Sex (male vs. female)	−6.64	83.63	0.938	0.046
Hcy	12	Terminal weight	−5.57	21.68	0.803	0.046
Met	12	Sex (male vs. female)	82.26	941.32	0.932	0.056
Met	12	Terminal weight	69.39	244.00	0.783	0.056
Cys	10	Sex (male vs. female)	5.12	6.07	0.426	0.107
Cys	10	Terminal weight	−1.54	1.73	0.403	0.107

## Data Availability

Raw mass spectrometry metabolomics data generated with this study are available at the public data repository MassIVE, maintained at UCSD (https://massive.ucsd.edu/ProteoSAFe/static/massive.jsp?redirect=auth, was accessed on 3 September 2026) under the deposition number MSV000101816. All other raw data supporting the conclusions of this article will be made available from the corresponding authors upon reasonable request.

## Supplementary Materials

The following supporting information can be downloaded at https://www.mdpi.com/article/10.3390/antiox15091162/s1, Table S1: Control Diet Formula (Envigo); Table S2: Hyperhomocysteinemia-inducing diet Formula (Envigo); Supplementary Materials S1: Method Development Data; Supplementary Materials S2: Intraday Validation Data for each replicate by QC level; Supplementary Materials S3: Interday Validation Data for each replicate by QC level and by day; Supplementary Materials S4: Individual replicates of homocysteine, methionine, and cysteine values; Supplementary Figure S1: Representative extracted chromatograms from mouse plasma samples.
