# Supplementary material for: Diet-Induced Hyperhomocysteinemia in C57BL/6 Mice: Simultaneous Evaluation of Plasma Homocysteine, Methionine, and Cysteine by LC-MS/MS and Assessment of Associated Oxidative Stress in Brain Homogenates
Source: Antioxidants (Basel). 2026 Sep 11;15(9):1162. doi: 10.3390/antiox15091162 (PMC13603794; doi:10.3390/antiox15091162)
Supplement: Supplementary file 1 [file antioxidants-15-01162-s001.zip › antioxidants-4375343-supplementary.pdf]

Supplementary Table S1. Control Diet Formula (Envigo)

**Control Diet, Formula TD.220641**	**g/kg**
Casein, "Vitamin-Free" Test	195.0
DL-Methionine	3.0
Sucrose	335.1
Corn Starch	300.0
Soybean Oil	70.0
Cellulose	40.0
Mineral Mix, AIN-93G-MX (94046)	35.0
Calcium Phosphate, dibasic	4.0
Choline Bitartrate	2.75
Vitamin Mix, AIN-93-VX (94047)	15.0
Vitamin K1, phylloquinone	0.002
Pink Food Color	0.1

Supplementary Table S2. Hyperhomocysteinemia-inducing Diet Formula (Envigo)

**Hyperhomocysteinemia Diet, Formula TD.220642**	**g/kg**
Casein, "Vitamin-Free" Test	195.0
DL-Methionine	7.7
Sucrose	345.1
Corn Starch	300.0
Soybean Oil	70.0
Cellulose	40.0
Mineral Mix, AIN-93G-MX (94046)	35.0
Calcium Phosphate, dibasic	4.0
Choline Bitartrate	2.75
Niacin	0.045
Calcium Pantothenate	0.024
Thiamin (81%)	0.009
Riboflavin	0.009
Biotin	0.0003
Vitamin E, DL-alpha tocopheryl acetate (500 IU/g)	0.225
Vitamin A Palmitate (500,000 IU/g)	0.012
Vitamin D3, cholecalciferol (500,000 IU/g)	0.003
Vitamin K1, phylloquinone	0.002
Yellow Food Color	0.1

Supplementary Materials S1: Method Development Data

Supplementary Materials S1.1. Preliminary method development for total homocysteine measurement

S1.1.1. Rationale for aqueous surrogate calibration

Total homocysteine is an endogenous analyte, making preparation of a truly analyte-free mouse serum or plasma calibration matrix challenging. During preliminary method development, different calibration matrix approaches were evaluated to reduce endogenous background contribution at the low concentration range. Commercial mouse serum was initially assessed as a calibration matrix but showed background interference that affected low-end quantification and produced unreliable or negative calculated concentrations in some control mouse serum samples.

Because of these observations, non-plasma surrogate calibration approaches were explored. Although BSA solution was evaluated during preliminary development, the final method used 0.1% formic acid in water as the surrogate calibration matrix for preparation of calibration standards. This approach minimized endogenous background contribution and allowed consistent low-end calibration for exploratory quantification of total homocysteine in limited-volume mouse samples.

These preliminary optimization experiments were performed during development of the total homocysteine assay. Methionine and cysteine were incorporated into the LC-MS/MS workflow later; therefore, the optimization data presented here support the rationale for the homocysteine component of the method and low-volume sample processing, but should not be interpreted as complete optimization or full validation of all three analytes.

S1.1.2. Preliminary matrix comparison during total homocysteine method development

Commercial mouse serum and BSA solution were evaluated during preliminary total homocysteine method development to assess the effect of matrix background on low-end quantification. Extraction and instrumental conditions were kept constant during this comparison. Both approaches produced acceptable linearity, with R^2 values greater than 0.995, and no meaningful retention-time shift was observed for homocysteine or homocysteine-d4.

However, commercial mouse serum showed evidence of endogenous/background contribution that affected low-end calculations. In contrast, the BSA comparison reduced low-end problems and avoided negative calculated values in control mouse serum samples. These observations supported the decision to avoid serum-based calibration for the final assay. The final calibration matrix used in the manuscript was 0.1% formic acid in water, not BSA solution.

Preliminary matrix comparison during total homocysteine method development.

Effect on Linearity

Using Commercial Mouse Serum

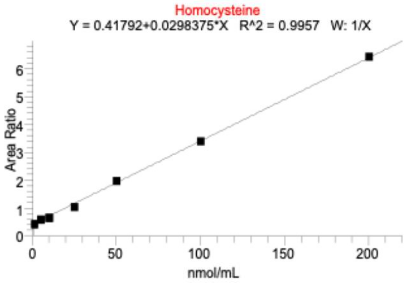

Slope = 0.0298375 or 0.03
Y-intercept = 0.41792 or 0.4
R^2 = 0.9957

Using BSA Solution

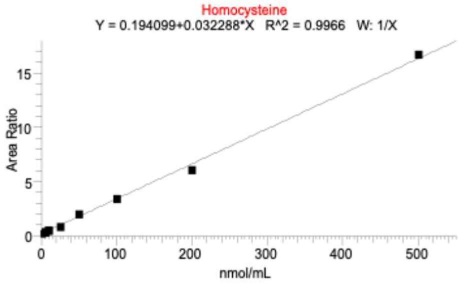

Slope = 0.032288 or 0.03
Y-intercept = 0.194099 or 0.2 **Y-intercept was reduced!**
R^2 = 0.9966

Effect on Retention Time

Using Commercial Mouse Serum

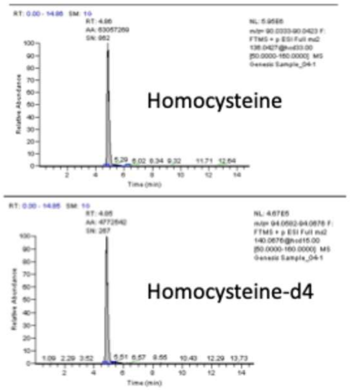

Homocysteine RT = 4.86 mins
Homocysteine-d4 = 4.85 mins

Using BSA Solution

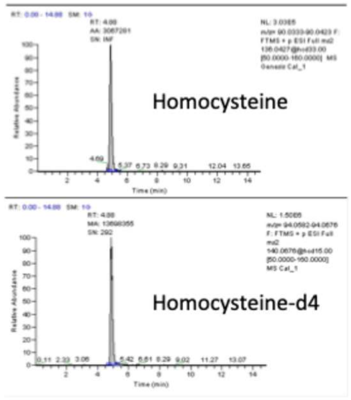

Homocysteine RT = 4.88 mins
Homocysteine-d4 = 4.88 mins

NO SIGNIFICANT EFFECT OR SHIFT IN RT

Effect on Measurement of Total HCys Concentrations

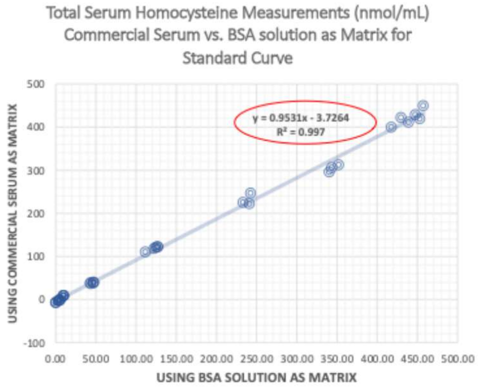

Slope < 1.0 and negative y-intercept indicates measurements using commercial serum are lower than using BSA solution as matrix.

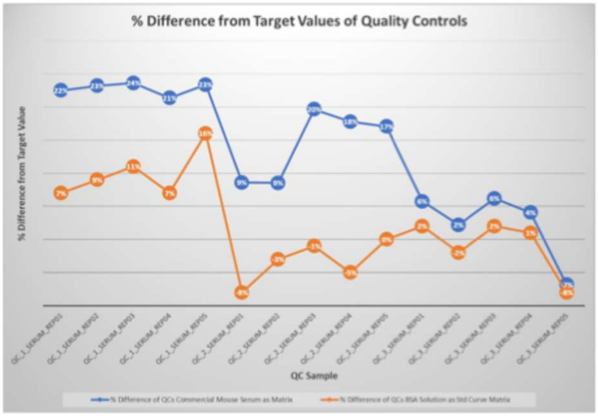

Higher % Difference Values in QCs observed when Commercial Serum is used as matrix (blue).

Effect on Measurement of Total HCys Concentrations

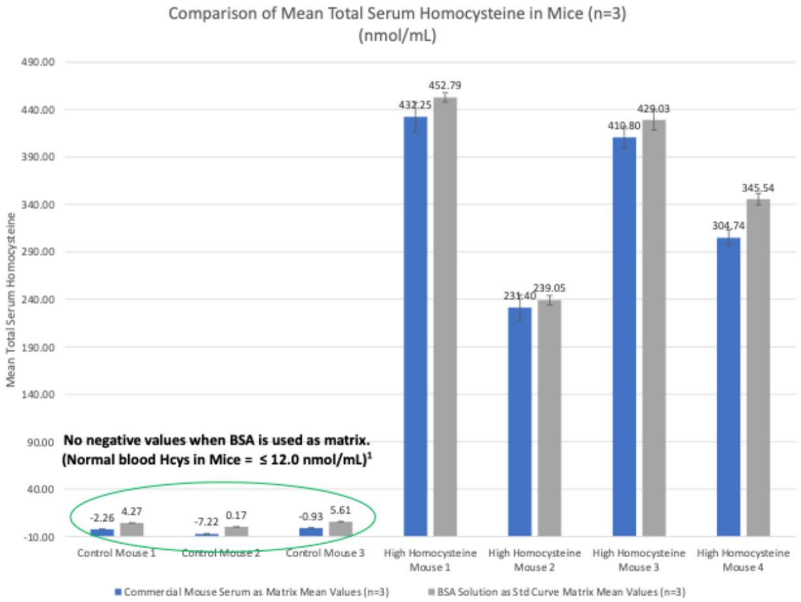

[1] Ernest S, Hosack A, O'Brien WE, Rosenblatt DS, Nadeau JH. Homocysteine levels in A/J and C57BL/6J mice: genetic, diet, gender, and parental effects. Physiol Genomics. 2005;21(2):404-410. doi:10.1152/physiolgenomics.00199.2004

Supplemental Slides (QC)

	Total Hcys Values (nmol/mL)		% Difference of QCs	
Samples in Serum	Commercial Mouse Serum as Matrix	BSA Solution as Std Curve Matrix	Commercial Mouse Serum as Matrix	BSA Solution as Std Curve Matrix
QC_1_serum_rep01	8.597	9.80	22%	7%
QC_1_serum_rep02	8.689	9.86	23%	9%
QC_1_serum_rep03	8.893	9.89	24%	11%
QC_1_serum_rep04	8.561	9.71	21%	7%
QC_1_serum_rep05	9.255	9.87	23%	16%
QC_2_serum_rep01	36.617	43.43	9%	-8%
QC_2_serum_rep02	38.753	43.41	9%	-3%
QC_2_serum_rep03	39.524	47.86	20%	-1%
QC_2_serum_rep04	38.155	47.11	18%	-5%
QC_2_serum_rep05	39.817	46.82	17%	0%
QC_3_serum_rep01	122.74	126.89	6%	2%
QC_3_serum_rep02	117.963	122.64	2%	-2%
QC_3_serum_rep03	122.015	127.43	6%	2%
QC_3_serum_rep04	120.722	124.90	4%	1%
QC_3_serum_rep05	110.394	111.81	-7%	-8%

Commercial mouse serum and BSA solution were evaluated as calibration matrix approaches during preliminary total homocysteine method development. Both approaches showed acceptable linearity and no meaningful retention-time shift. Commercial mouse serum showed low-end background contribution affecting calculated concentrations in control mouse serum samples, supporting the use of a non-plasma surrogate calibration approach. The final method used 0.1% formic acid in water as the surrogate calibration matrix.

S1.1.3. Sample-volume optimization for total homocysteine measurement

To assess whether total homocysteine measurement could be adapted for limited mouse serum/plasma availability, sample input volumes of 50 µL, 25 µL, and 10 µL serum were compared. Solvent volumes were scaled proportionally, while chromatographic and mass spectrometric acquisition parameters were kept constant.

The 10 µL condition preserved acceptable analytical performance for total homocysteine. Calibration linearity remained high across the tested volumes, with R^2 values of 0.9966, 0.9985, and 0.9974 for 50 µL, 25 µL, and 10 µL serum, respectively. The calculated LOD/LOQ values were also comparable across volumes, including an LOD of 0.54 nmol/mL and LOQ of 1.63 nmol/mL for the 10 µL condition. Retention times for homocysteine and homocysteine-d4 remained stable across the tested volumes, with no significant retention-time shift observed.

Accuracy values were within ±20% across the tested volumes, and precision values remained below 15% RSD. For the 10 µL serum condition, %RSD values were 4.87%, 4.97%, and 1.60% at QC1, QC2, and QC3, respectively. These data supported the feasibility of using 10 µL serum/plasma input for total homocysteine measurement in mouse samples.

Sample-volume comparison during total homocysteine method development.

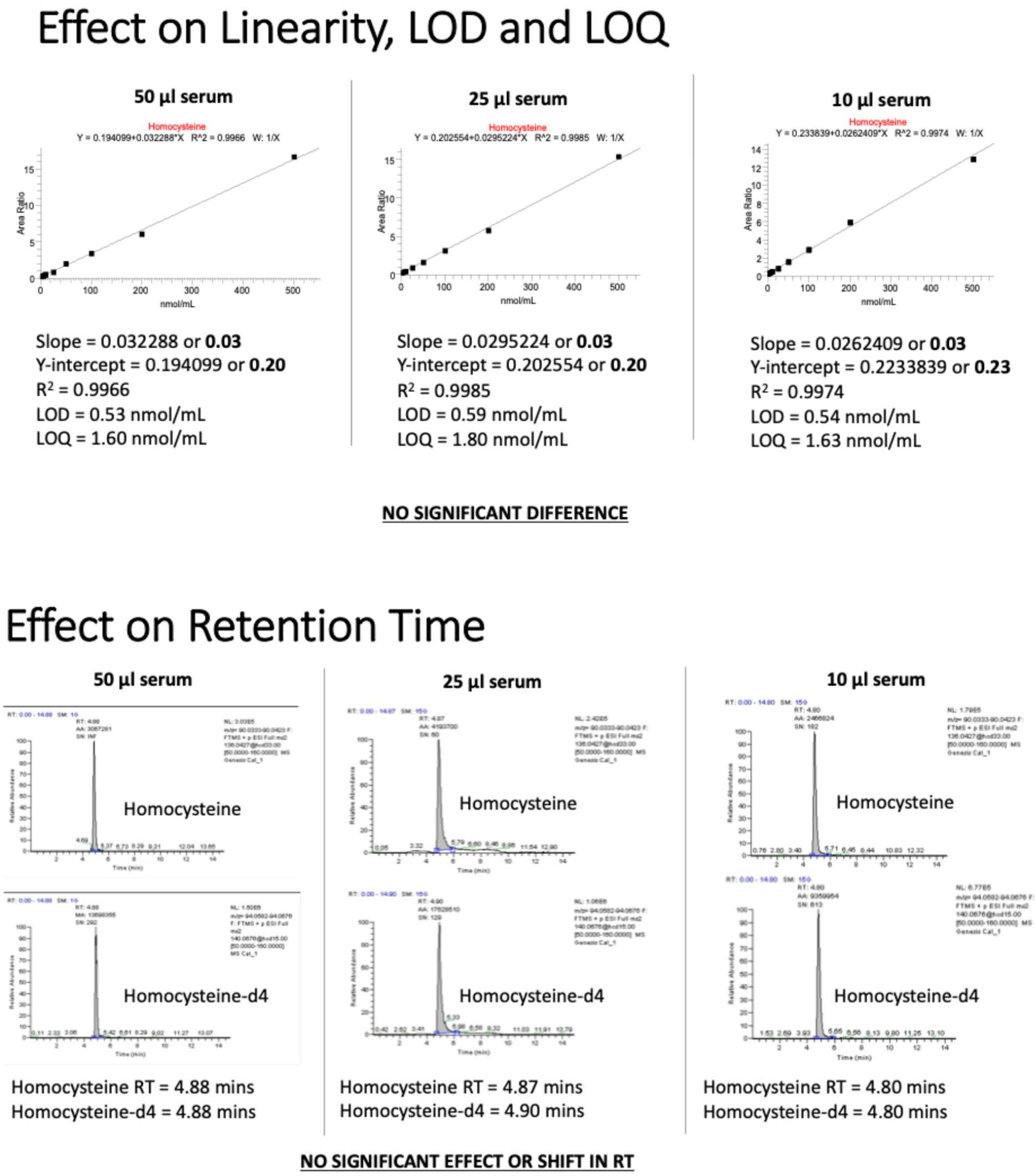

Effect on Accuracy of Total HCys Concentrations Measurements

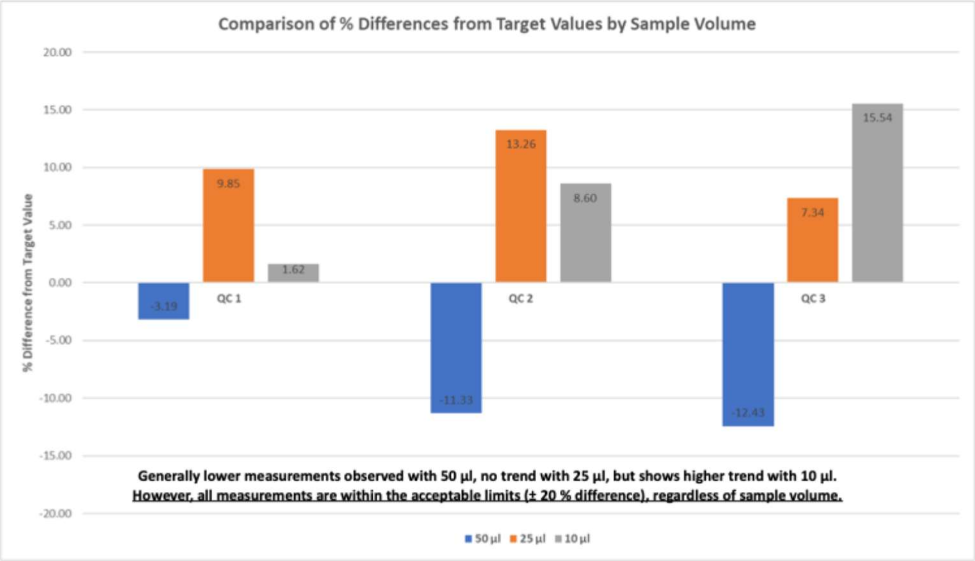

Note: For 50 µl, n = 3; for 25 and 10 µl, n = 5.

Effect on Precision of Total HCys Concentrations Measurements

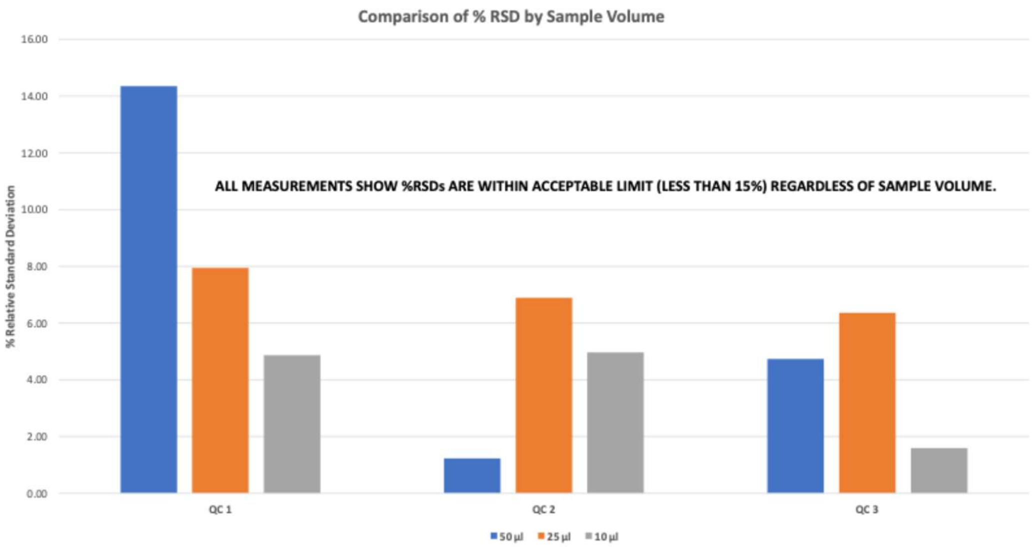

Note: For 50 µl, n = 3; for 25 and 10 µl, n = 5.

Supplemental Slide (Raw Data)

	Total Hcys Values (nmol/mL)		
Samples in Serum	50 µl	25 µl	10 µl
QC_1_rep01	8.98	8.31	7.87
QC_1_rep02	7.43	9.52	8.79
QC_1_rep03	6.83	8.87	8.00
QC_1_rep04		9.37	7.81
QC_1_rep05		7.87	8.18
QC_2_rep01	35.34	40.04	46.19
QC_2_rep02	35.11	47.56	40.16
QC_2_rep03	35.96	46.65	44.00
QC_2_rep04		44.92	43.33
QC_2_rep05		47.35	43.53
QC_3_rep01	107.29	141.25	140.14
QC_3_rep02	108.59	121.98	136.05
QC_3_rep03	99.39	125.75	140.28
QC_3_rep04		122.31	136.41
QC_3_rep05		132.75	140.37

	Mean	Mean	Mean
QC 1	7.75	8.79	8.13
QC 2	35.47	45.31	43.44
QC 3	105.09	128.81	138.65
	Std Dev	Std Dev	Std Dev
QC 1	1.111225	0.698397093	0.396092161
QC 2	0.44	3.12	2.16
QC 3	4.98	8.19	2.22
	% Difference from Target Value	% Difference from Target Value	% Difference from Target Value
QC 1	-3.19	9.85	1.62
QC 2	-11.33	13.26	8.60
QC 3	-12.43	7.34	15.54
	% RSD	% RSD	% RSD
QC 1	14.35	7.95	4.87
QC 2	1.23	6.89	4.97
QC 3	4.74	6.36	1.60

Total homocysteine measurement was compared using 50 µL, 25 µL, and 10 µL mouse serum input volumes. Solvent volumes were scaled proportionally, and LC-MS/MS acquisition conditions were kept constant. The 10 µL condition maintained acceptable linearity, LOD/LOQ, retention-time stability, accuracy, and precision, supporting the feasibility of low-volume sample processing.

S1.1.4. Scope and limitations of the optimization data

The matrix and sample-volume comparison experiments were performed during preliminary development of the total homocysteine assay. These data support the rationale for avoiding serum-based calibration, using a non-plasma surrogate calibration approach, and reducing sample volume for mouse serum/plasma analysis.

However, methionine and cysteine were added to the LC-MS/MS workflow after the initial homocysteine optimization experiments. Therefore, these supplementary data should be interpreted as method-development support for total homocysteine measurement and low-volume sample processing, rather than as complete optimization or full validation of the final simultaneous homocysteine, methionine, and cysteine panel.

Formal matrix-effect, extraction recovery, and stability experiments were not performed for the final three-analyte workflow. Accordingly, the method is best described as a fit-for-purpose LC-MS/MS workflow for exploratory preclinical plasma profiling, rather than as a fully validated regulatory bioanalytical assay.

Supplemental Materials S2. Intraday Validation Data for each replicate by QC Level.

			Cysteine	Methionine	Homocysteine	Cysteine	Methionine	Homocysteine	Cysteine	Methionine	Homocysteine
QC Sample	QC Level	Target Value	Area Response	Area Response	Area Response	Conc (μmol/L)	Conc (μmol/L)	Conc (μmol/L)	%Diff	%Diff	%Diff
QC1-01	1	8	0.5119	0.2154	0.3486	7.51	8.97	6.80	-6.10	12.18	-14.95
QC1-02	1	8	0.4991	0.2148	0.3476	7.34	8.95	6.78	-8.26	11.88	-15.20
QC1-03	1	8	0.4930	0.2076	0.3532	7.26	8.65	6.89	-9.29	8.18	-13.87
QC1-04	1	8	0.4859	0.2107	0.3468	7.16	8.78	6.77	-10.50	9.75	-15.36
QC1-05	1	8	0.4777	0.2134	0.3493	7.05	8.89	6.82	-11.90	11.15	-14.78
QC1-06	1	8	0.4935	0.2081	0.3511	7.26	8.67	6.85	-9.21	8.39	-14.35
QC1-07	1	8	0.5025	0.2090	0.3519	7.39	8.71	6.87	-7.68	8.86	-14.16
QC1-08	1	8	0.4781	0.2133	0.3459	7.05	8.89	6.75	-11.83	11.06	-15.58
QC1-09	1	8	0.4969	0.2115	0.3526	7.31	8.81	6.88	-8.64	10.16	-14.01
QC1-10	1	8	0.4895	0.2141	0.3401	7.21	8.92	6.64	-9.89	11.49	-16.97
QC2-01	2	40	2.8772	0.9004	1.8058	39.63	37.12	34.33	-0.94	-7.19	-14.18
QC2-02	2	40	2.9165	0.9118	1.7839	40.16	37.60	33.92	0.40	-6.01	-15.21
QC2-03	2	40	2.7622	0.9049	1.7618	38.06	37.31	33.50	-4.84	-6.72	-16.25
QC2-04	2	40	2.8332	0.9188	1.7601	39.03	37.88	33.47	-2.43	-5.29	-16.33
QC2-05	2	40	2.7554	0.9227	1.7578	37.97	38.04	33.42	-5.07	-4.90	-16.44
QC2-06	2	40	2.8574	0.9177	1.7748	39.36	37.84	33.74	-1.61	-5.41	-15.64
QC2-07	2	40	2.8060	0.9156	1.7993	38.66	37.75	34.21	-3.35	-5.62	-14.48
QC2-08	2	40	2.8037	0.9142	1.7645	38.63	37.69	33.55	-3.43	-5.77	-16.12
QC2-09	2	40	2.7397	0.9146	1.7554	37.76	37.71	33.38	-5.60	-5.73	-16.55
QC2-10	2	40	2.7125	0.9166	1.7832	37.39	37.79	33.90	-6.53	-5.52	-15.24
QC3-01	3	120	9.0852	2.4558	5.2467	123.91	101.05	99.33	3.26	-15.79	-17.23
QC3-02	3	120	9.1189	2.4545	5.3826	124.37	101.00	101.89	3.64	-15.84	-15.09
QC3-03	3	120	9.0243	2.4527	5.4175	123.08	100.92	102.55	2.57	-15.90	-14.54
QC3-04	3	120	9.0986	2.4802	5.2225	124.09	102.05	98.87	3.41	-14.96	-17.61
QC3-05	3	120	9.0081	2.4471	5.1623	122.86	100.69	97.73	2.38	-16.09	-18.56
QC3-06	3	120	7.5426	2.4470	5.3451	102.97	100.69	101.19	-14.20	-16.09	-15.68
QC3-07	3	120	7.7880	2.4679	5.2681	106.30	101.55	99.73	-11.42	-15.38	-16.89
QC3-08	3	120	7.7135	2.4375	5.2308	105.28	100.30	99.03	-12.26	-16.42	-17.48
QC3-09	3	120	7.5438	2.4603	5.2346	102.98	101.23	99.10	-14.18	-15.64	-17.42
QC3-10	3	120	7.5480	2.3943	5.2563	103.04	98.52	99.51	-14.14	-17.90	-17.08

Supplementary Materials S3. Interday Validation Data for each replicate by QC Level and by day.

	Day 1			Day 2			Day 3		
	Cysteine	Methionine	Homocysteine	Cysteine	Methionine	Homocysteine	Cysteine	Methionine	Homocysteine
QC Sample	Conc (μmol/L)	Conc (μmol/L)	Conc (μmol/L)	Conc (μmol/L)	Conc (μmol/L)	Conc (μmol/L)	Conc (μmol/L)	Conc (μmol/L)	Conc (μmol/L)
QC1-01	7.51	8.97	6.80	9.47	7.80	7.37	11.06	7.52	7.37
QC1-02	7.34	8.95	6.78	9.25	7.78	7.34	10.80	7.50	7.35
QC1-03	7.26	8.65	6.89	9.15	7.47	7.46	10.67	7.19	7.47
QC1-04	7.16	8.78	6.77	9.02	7.60	7.33	10.53	7.32	7.33
QC1-05	7.05	8.89	6.82	8.88	7.72	7.38	10.36	7.44	7.38
QC1-06	7.26	8.67	6.85	9.15	7.49	7.42	10.69	7.21	7.42
QC1-07	7.39	8.71	6.87	9.31	7.53	7.44	10.87	7.25	7.44
QC1-08	7.05	8.89	6.75	8.89	7.71	7.31	10.37	7.43	7.31
QC1-09	7.31	8.81	6.88	9.21	7.64	7.45	10.75	7.36	7.46
QC1-10	7.21	8.92	6.64	9.09	7.74	7.19	10.60	7.47	7.18
QC2-01	39.63	37.12	34.33	50.27	36.47	38.13	59.69	36.32	39.30
QC2-02	40.16	37.60	33.92	50.95	36.95	37.67	60.50	36.80	38.82
QC2-03	38.06	37.31	33.50	48.29	36.66	37.20	57.33	36.51	38.33
QC2-04	39.03	37.88	33.47	49.51	37.25	37.17	58.79	37.09	38.30
QC2-05	37.97	38.04	33.42	48.17	37.41	37.12	57.19	37.25	38.25
QC2-06	39.36	37.84	33.74	49.93	37.20	37.48	59.28	37.05	38.62
QC2-07	38.66	37.75	34.21	49.04	37.11	37.99	58.23	36.96	39.15
QC2-08	38.63	37.69	33.55	49.00	37.05	37.26	58.18	36.90	38.39
QC2-09	37.76	37.71	33.38	47.90	37.07	37.07	56.87	36.91	38.19
QC2-10	37.39	37.79	33.90	47.43	37.15	37.65	56.31	37.00	38.80
QC3-01	123.91	101.05	99.33	157.35	101.59	110.77	187.32	101.71	114.69
QC3-02	124.37	101.00	101.89	157.93	101.53	113.64	188.02	101.65	117.67
QC3-03	123.08	100.92	102.55	156.30	101.45	114.38	186.07	101.58	118.43
QC3-04	124.09	102.05	98.87	157.58	102.60	110.26	187.60	102.73	114.16
QC3-05	122.86	100.69	97.73	156.02	101.22	108.99	185.74	101.34	112.84
QC3-06	102.97	100.69	101.19	130.74	101.21	112.85	155.61	101.34	116.85
QC3-07	106.30	101.55	99.73	134.98	102.09	111.22	160.65	102.22	115.16
QC3-08	105.28	100.30	99.03	133.69	100.82	110.44	159.12	100.94	114.34
QC3-09	102.98	101.23	99.10	130.77	101.77	110.51	155.64	101.90	114.43
QC3-10	103.04	98.52	99.51	130.84	99.01	110.97	155.72	99.12	114.90

Supplementary Materials S4. Individual replicates of Homocysteine, Methionine and Cysteine values *

Analyte	Diet and sex	Replicate	Concentration (μmol/L)
Homocysteine	Control, Female	rep01	2.86
		rep02	2.89
		rep03	2.92
		rep01	1.38
		rep02	1.42
		rep03	1.38
		rep01	1.20
		rep02	1.25
		rep03	1.29
		rep01	8.31
		rep02	8.18
		rep03	8.23
		rep01	7.25
		rep02	7.71
		rep03	7.83
	HHcy, Female	rep01	159.50
		rep02	161.21
		rep03	163.87
		rep01	110.42
		rep02	110.16
		rep03	110.83
		rep01	203.83
		rep02	207.27
		rep03	211.64
		rep01	245.90
		rep02	243.88
		rep03	246.55
		rep01	230.14
		rep02	231.99
		rep03	238.26
		rep01	194.78
		rep02	190.63
		rep03	195.64
		rep01	262.24
		rep02	274.13
		rep03	267.73

Homocysteine	Control, Male	rep01	6.53
		rep02	6.51
		rep03	6.58
		rep01	3.54
		rep02	3.61
		rep03	3.55
		rep01	2.75
		rep02	2.77
		rep03	2.90
		rep01	4.94
		rep02	4.91
		rep03	5.03
		rep01	5.45
		rep02	5.47
		rep03	5.44
		rep01	3.38
		rep02	3.36
		rep03	3.44
		rep01	2.25
		rep02	2.32
		rep03	2.43
		rep01	5.38
		rep02	5.40
		rep03	5.52
		rep01	3.60
		rep02	3.60
		rep03	3.63
	HHcy, Male	rep01	137.80
		rep02	140.40
		rep03	141.05
		rep01	84.33
		rep02	85.92
		rep03	84.73
		rep01	*25.97*
		rep02	*26.83*
		rep03	*26.60*
		rep01	312.74
		rep02	310.05
		rep03	313.37
		rep01	183.71
		rep02	191.33

		rep03	194.15
		rep01	160.15
		rep02	162.66
		rep03	161.84

Analyte	Diet and sex	Replicate	Concentration (μmol/L)
Methionine	Control, Female	rep01	212.11
		rep02	247.47
		rep03	266.57
		rep01	127.28
		rep02	134.67
		rep03	138.65
		rep01	59.73
		rep02	56.47
		rep03	54.96
		rep01	438.48
		rep02	452.95
		rep03	477.68
		rep01	308.48
		rep02	345.56
		rep03	358.19
	HHcy, Female	rep01	856.46
		rep02	889.51
		rep03	915.91
		rep01	1296.26
		rep02	1332.83
		rep03	1361.30
		rep01	1031.93
		rep02	1068.54
		rep03	1103.57
		rep01	430.22
		rep02	446.14
		rep03	457.72
		rep01	473.71
		rep02	498.74
		rep03	508.42
		rep01	2111.20
		rep02	2124.41
		rep03	2171.76
		rep01	1129.73
		rep02	1218.22

**Methionine**		rep03	1214.24
	**Control, Male**	rep01	197.91
		rep02	202.67
		rep03	201.20
		rep01	136.96
		rep02	144.56
		rep03	144.60
		rep01	464.93
		rep02	470.60
		rep03	483.36
		rep01	379.56
		rep02	374.58
		rep03	368.52
		rep01	233.29
		rep02	235.01
		rep03	238.09
		rep01	168.22
		rep02	171.36
		rep03	167.88
		rep01	74.60
		rep02	82.44
		rep03	83.63
		rep01	222.60
		rep02	234.22
		rep03	238.83
		rep01	143.25
		rep02	145.39
		rep03	141.64
	**HHcy, Male**	rep01	2482.99
		rep02	2595.33
		rep03	2592.38
		rep01	315.92
		rep02	331.88
		rep03	326.95
		rep01	*7.94*
		rep02	*11.00*
		rep03	*11.45*
		rep01	441.46
		rep02	446.73
		rep03	456.12
		rep01	1527.81

		rep02	1595.15
		rep03	1664.75
		rep01	2008.82
		rep02	2061.92
		rep03	2039.98

Analyte	Sample ID	Replicate	Concentration (μmol/L)
Cysteine	Control, Female	rep01	11.95
		rep02	12.26
		rep03	12.60
		rep01	9.23
		rep02	9.39
		rep03	9.47
		rep01	15.68
		rep02	15.86
		rep03	16.60
		rep01	14.63
		rep02	14.15
		rep03	14.51
		rep01	11.87
		rep02	12.51
		rep03	12.51
	HHcy, Female	rep01	3.22
		rep02	3.22
		rep03	3.22
		rep01	2.13
		rep02	2.13
		rep03	2.10
		rep01	8.89
		rep02	9.03
		rep03	8.80
		rep01	5.45
		rep02	5.50
		rep03	5.50
		rep01	6.09
		rep02	5.88
		rep03	6.02
		rep01	19.35
		rep02	19.23
		rep03	19.95
		rep01	7.36

Cysteine		rep02	7.68
		rep03	7.67
	Control, Male	rep01	24.31
		rep02	24.27
		rep03	24.84
		rep01	22.68
		rep02	23.40
		rep03	22.19
		rep01	19.25
		rep02	18.51
		rep03	19.55
		rep01	28.24
		rep02	25.58
		rep03	27.24
		rep01	25.64
		rep02	24.45
		rep03	24.02
		rep01	25.42
		rep02	25.52
		rep03	25.96
		rep01	23.73
		rep02	23.07
		rep03	23.34
		rep01	29.50
		rep02	30.11
		rep03	30.55
		rep01	27.04
		rep02	27.50
		rep03	26.95
	HHcy, Male	rep01	23.34
		rep02	23.57
		rep03	23.20
		rep01	8.29
		rep02	8.14
		rep03	7.90
		rep01	*63.66*
		rep02	*62.99*
		rep03	*62.86*
		rep01	43.76
		rep02	41.91
		rep03	42.45

		rep01	6.68
		rep02	6.94
		rep03	7.10
		rep01	9.87
		rep02	9.76
		rep03	9.82

*Red font indicates analyte concentration in a mouse that exhibited signs of poor health and was excluded from the study; blue font indicates values identified as outliers via ROUT (Q=10 %) analysis (GraphPad Prism) and excluded from the data set.

Supplementary Figure S1: Representative extracted chromatograms from mouse plasma samples

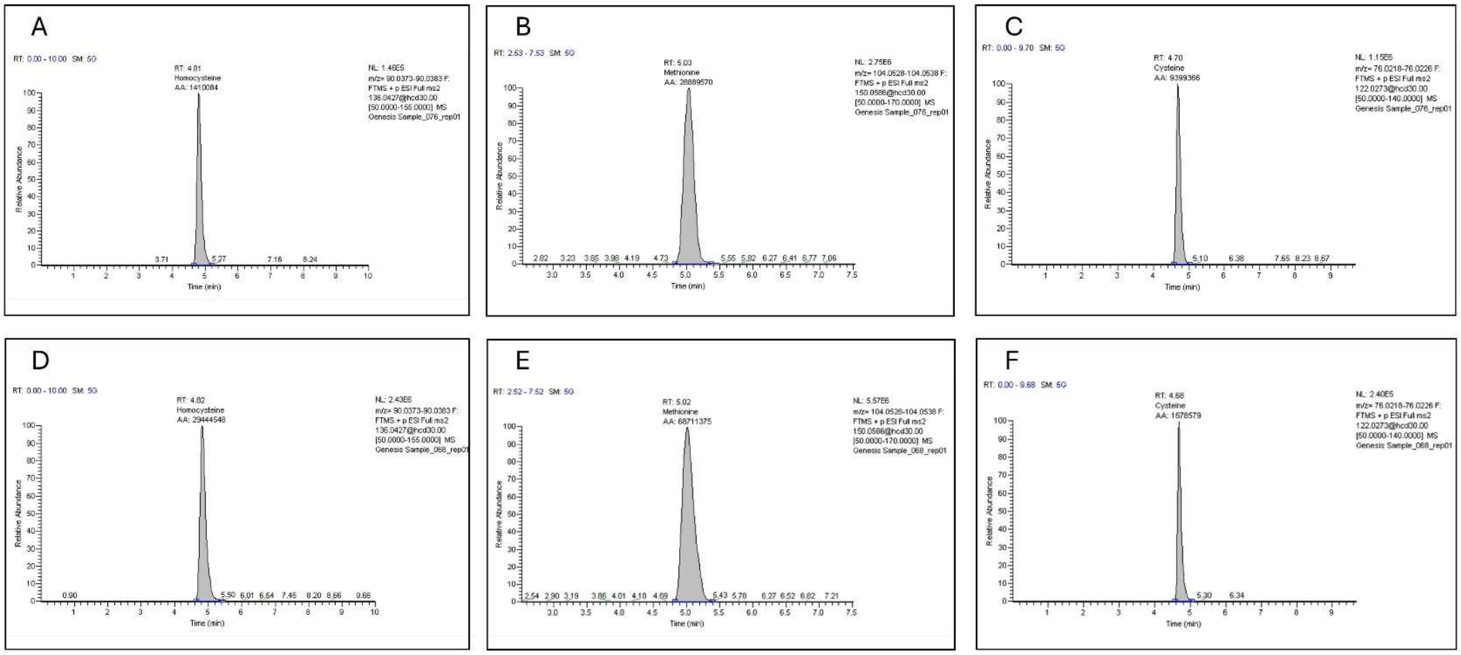

Supplementary Figure S1. **Representative extracted ion chromatograms from mouse plasma samples.** The figure illustrates representative extracted ion chromatograms (XICs) of Hcy, Met, and Cys for a non-outlier control-diet sample, Sample_076, and a non-outlier HHcy-diet sample, Sample_068, both included in the final analysis. Sample_076 is shown in panels A–C: (**A**) Hcy XIC as 136 m/z → 90 m/z, (**B**) Met XIC as 150 m/z → 104 m/z, (**C**) Cys XIC as 122 m/z → 76 m/z). This control-diet sample showed average measured concentrations of Hcy 5.46 µmol/L, Met 235.47 µmol/L, and Cys 24.71 µmol/L. Sample_068 is shown in panels D–F: (**D**) Hcy XIC as 136 m/z → 90 m/z, (**E**) Met XIC as 150 m/z → 104 m/z, (**F**) Cys XIC as 122 m/z → 76 m/z). This HHcy-diet Sample_068 showed average measured concentrations of Hcy 207.59 µmol/L, Met 1068.02 µmol/L, and Cys 8.91 µmol/L.
